# Repair of acute respiratory distress syndrome by stromal cell administration (REALIST): a structured study protocol for an open-label dose-escalation phase 1 trial followed by a randomised, triple-blind, allocation concealed, placebo-controlled phase 2 trial

**DOI:** 10.1186/s13063-022-06220-0

**Published:** 2022-05-13

**Authors:** Ellen Gorman, Manu Shankar-Hari, Phil Hopkins, William S. Tunnicliffe, Gavin D. Perkins, Jonathan Silversides, Peter McGuigan, Colette Jackson, Roisin Boyle, Jamie McFerran, Cliona McDowell, Christina Campbell, Margaret McFarland, Jon Smythe, Jacqui Thompson, Barry Williams, Gerard Curley, John G. Laffey, Mike Clarke, Daniel F. McAuley, Cecilia O’Kane

**Affiliations:** 1 Wellcome-Wolfson Institute for Experimental Medicine, Queen’s University, Belfast, UK; 2 Guy’s and St Thomas’ NHS Foundation Trust London, London, UK; 3 School of Immunology and Microbial Sciences, King’s College London, London, UK; 4 Centre for Inflammation Research, The University of Edinburgh, Edinburgh, UK; 5 Kings Trauma Centre, King’s College London, London, UK; 6 Queen Elizabeth Hospital Birmingham, Birmingham, UK; 7 Warwick Clinical Trials Unit, Warwick Medical School, University of Warwick, Coventry, UK; 8 University Hospitals Birmingham, Birmingham, UK; 9 Department of Critical Care, Belfast Health and Social Care Trust, Belfast, UK; 10 Northern Ireland Clinical Trials Unit, Belfast, UK; 11 NHS Blood and Transplant Service, Oxford, UK; 12 NHS Blood and Transplant Service, Birmingham, UK; 13 Independent Patient and Public Representative, Sherborne, UK; 14 Royal College of Surgeons in Ireland, Dublin, Ireland; 15 Regenerative Medicine Institute (REMEDI) at CÚRAM Centre for Research in Medical Devices, National University of Ireland, Galway, Ireland

**Keywords:** Acute respiratory distress syndrome, Mesenchymal stromal cells, MSCs, Mesenchymal stem cells, Clinical trial, Protocol, COVID-19

## Abstract

**Background:**

Mesenchymal stromal cells (MSCs) may be of benefit in ARDS due to immunomodulatory and reparative properties. This trial investigates a novel CD362 enriched umbilical cord derived MSC product (REALIST ORBCEL-C), produced to Good Manufacturing Practice standards, in patients with moderate to severe ARDS due to COVID-19 and ARDS due to other causes.

**Methods:**

Phase 1 is a multicentre open-label dose-escalation pilot trial. Patients will receive a single infusion of REALIST ORBCEL-C (100 × 10^6^ cells, 200 × 10^6^ cells or 400 × 10^6^ cells) in a 3 + 3 design. Phase 2 is a multicentre randomised, triple blind, allocation concealed placebo-controlled trial. Two cohorts of patients, with ARDS due to COVID-19 or ARDS due to other causes, will be recruited and randomised 1:1 to receive either a single infusion of REALIST ORBCEL-C (400 × 10^6^ cells or maximal tolerated dose in phase 1) or placebo. Planned recruitment to each cohort is 60 patients. The primary safety outcome is the incidence of serious adverse events. The primary efficacy outcome is oxygenation index at day 7. The trial will be reported according to the Consolidated Standards for Reporting Trials (CONSORT 2010) statement.

**Discussion:**

The development and manufacture of an advanced therapy medicinal product to Good Manufacturing Practice standards within NHS infrastructure are discussed, including challenges encountered during the early stages of trial set up. The rationale to include a separate cohort of patients with ARDS due to COVID-19 in phase 2 of the trial is outlined.

**Trial registration:**

ClinicalTrials.gov NCT03042143. Registered on 3 February 2017. EudraCT Number 2017-000584-33

**Supplementary Information:**

The online version contains supplementary material available at 10.1186/s13063-022-06220-0.

## Administrative information

Note: the numbers in curly brackets in this protocol refer to SPIRIT checklist item numbers. The order of the items has been modified to group similar items (see http://www.equator-network.org/reporting-guidelines/spirit-2013-statement-defining-standard-protocol-items-for-clinical-trials/).

**Table:** 

Title {1}	Repair of Acute Respiratory Distress Syndrome by Stromal Cell Administration (REALIST): A structured study protocol for an open label dose escalation phase 1 trial followed by a randomised, triple-blind, allocation concealed, placebo controlled trial.
Trial registration {2a and 2b}.	ClinicalTrials.gov NCT03042143. Registered February 3^rd^ 2017, https://clinicaltrials.gov/ct2/show/NCT03042143 EudraCT Number 2017-000584-33
Protocol version {3}	6.0 30.09.20
Funding {4}	Wellcome Trust Health Innovation Challenge Fund [Reference 106939/Z/15/Z] Research and Development Division of the Public Health AgencyNorthern Ireland
Author details {5a}	**EG** Clinical Research Fellow, Wellcome-Wolfson Institute for Experimental Medicine, Queen’s University, Belfast **MSH** NIHR Clinician Scientist, Professor and Consultant in ICM, Guy’s and St Thomas’ NHS Foundation Trust London, School of Immunology and Microbial Sciences, King’s College London, and Centre for Inflammation Research, The University of Edinburgh, Edinburgh **PH** Research and Development lead in Critical Care, Kings Trauma Centre, King’s College London **WST** Consultant in Respiratory and Critical Care Medicine, Queen Elizabeth Hospital, Birmingham **GDP** Professor of Critical Care Medicine, University of Warwick and University Hospitals Birmingham **JS** Consultant in Critical Care, Belfast Health and Social Care Trust and Clinical Senior Lecturer, Wellcome-Wolfson Institute for Experimental Medicine **PMCG** Consultant in Intensive Care Medicine, Belfast Health and Social Care Trust **CJ** Clinical Trials Manager, Northern Ireland Clinical Trials Unit **RB** Clinical Trials Manager, Northern Ireland Clinical Trials Unit** JMCF** Trial Co-ordinator, Northern Ireland Clinical Trials Unit **CMCD** Head of Statistics, Northern Ireland Clinical Trials Unit** CC** Senior Biostatistician, Northern Ireland Clinical Trials Unit **MMCF** Lead Clinical Trials Pharmacist, Belfast Health and Social Care Trust** JS** Head of Cellular and Molecular Therapies, NHS Blood and Transplant Service** JT** Lead Translational Therapy Scientist, NHS Blood and Transplant Service **BW** Independent Patient and Public Representative **GC** Professor of Anaesthetics and Critical Care Medicine, Royal College of Surgeons in Ireland **JL** Professor of Anaesthesia and Critical Care Medicine, National University of Ireland, Galway **MC** Professor/Director of Northern Ireland Methodology Hub, School of Medicine, Dentistry and Biomedical Sciences, Queen’s University, Belfast and Director Northern Ireland Clinical Trials Unit **DFM** Professor of Critical Care Medicine, Wellcome-Wolfson Institute for Experimental Medicine, Queen’s University, Belfast **COK** Professor of Respiratory Medicine, Wellcome-Wolfson Institute for Experimental Medicine, Queen’s University, Belfast
Name and contact information for the trial sponsor {5b}	Belfast Health and Social Care Trust (BHSCT) The Royal Hospitals Grosvenor Road Belfast BT12 6BA Northern Ireland
Role of sponsor {5c}	The Belfast Health and Social Care Trust (BHSCT) will act as Sponsor for the trial and the Chief investigator will take overall responsibility for the conduct of the trial. The funders have no role in the study design, data acquisition, data analysis or manuscript preparation.

## Introduction

### Background and rationale {6a}

Acute respiratory distress syndrome (ARDS) is characterised by acute hypoxaemia and bilateral radiographic opacities [1]. Its mortality burden is high, between 35 and 45% depending on severity of hypoxia [2], and physical and psychological morbidity in survivors is considerable, with exercise limitation persisting at 5 years and psychological sequelae including anxiety, depression and post-traumatic stress disorder reported in 50% [3, 4]. In a pre-COVID-19 era, ARDS accounted for 10% of intensive care unit (ICU) admissions and 23% of patients requiring mechanical ventilation [2]. The global burden of ARDS has likely increased during the COVID-19 pandemic with the incidence of ARDS in hospitalised COVID-19 patients reported to be between 17 and 68% [5]. ARDS has significant economic burden, within the NHS the estimated cost per patient during hospital admission is £25 K (GBP) [6]. The complex pathogenesis of ARDS is initiated by an inflammatory insult and driven by immune activation and cytokine release. There is loss of integrity of the epithelial-endothelial barrier in the alveolar-capillary unit. Subsequently, production of an inflammatory exudate causes alveolar and interstitial oedema with loss of pulmonary compliance and impaired gas exchange [7]. Supportive care in the critical care environment is the mainstay of treatment for ARDS [8]. Despite numerous clinical trials, there are no therapeutic agents proven to be effective for ARDS [9]. When this trial was designed, there were no pharmacological treatments for COVID-19, although recent trials have demonstrated dexamethasone and IL-6 antagonism reduced 28-day mortality in mechanically ventilated patients with COVID-19 [10–13]. There remains a need for pharmacological treatments in ARDS, as well as to further improve outcomes in ARDS due to COVID-19.

Mesenchymal stromal cells (mesenchymal stem cells, MSCs) are an advanced therapy medicinal product (ATMP) proposed as a therapeutic agent in ARDS due to their immunomodulatory, reparative and antimicrobial properties [14]. They are a heterogenous cell population which can be isolated from various tissue sources (including bone marrow, adipose and umbilical cord tissue (UCT, Wharton’s Jelly)) [15]. MSCs have demonstrated considerable benefit in pre-clinical models of ARDS [16–20]. MSCs have been investigated in early phase clinical trials in ARDS. Matthay et al. have reported a phase 1 and phase 2 trial of a single infusion of bone-marrow derived MSCs in patients with moderate to severe ARDS [21, 22]. In the 60 patient phase 2 trial, no MSC-related safety events were reported; however, the trial was not powered for clinical outcomes [22]. MUSTARDS is a phase 1/2 trial of a single infusion of MultiStem (bone marrow-derived multipotent adult progenitor cells, BM-MAPC) in 36 patients with ARDS. This cellular product has similarities to MSCs but represents a subtype with distinct phenotypical and functional differences [23]. Similarly, this trial was not powered for clinical outcomes but results suggest MAPC were safe for use in patients with ARDS [24]. Umbilical cord (UC)- and adipose tissue (AT)-derived MSCs have also been investigated in small phase 1 trials in ARDS, and no safety concerns have been identified [25, 26].

There has been considerable interest in the use of MSCs for the treatment of COVID-19, with over 60 trials registered on clinicaltrials.gov during the COVID-19 pandemic (accessed April 2021). Case series and observational studies have not identified any safety concerns [27–32]. Shi et al. [33] have reported a randomised placebo-controlled trial of multiple doses of umbilical cord MSCs in COVID-19; however, patients were in a convalescent stage (median time from symptom onset—MSC group 45 days vs placebo group 47 days). The trial did not meet its primary outcome of reducing total lung lesions as proportion of the whole lung (MSC group vs placebo, −13.31%, 95% CI − 29.14 to 2.13%, *p* = 0.08) from baseline to day 28 [33]. Improvement in secondary outcomes, including in solid component lesions as a proportion of the whole lung and improved performance on a 6-m walk test at day 28 in the MSC-treated group, supported the reparative potential of MSC administration in COVID-19 induced lung disease [33]. Lanzoni et al. reported a small randomised controlled trial investigating administration of two intravenous infusions of UC derived MSCs (100 × 10^6^) or placebo control to patients with COVID-19 ARDS (randomised 1:1, *n* = 12 in each group). UC-MSCs were found to be safe, with no infusion-associated adverse events or serious adverse events related to MSC infusions [34]. Dilogo et al. reported a further small randomised controlled trial investigating administration of a single infusion of UC-derived MSCs (1 × 10^6^ MSCs/kg) or placebo control to critically ill patients with COVID-19 (randomised 1:1, *n* = 20 in each group) [35]. In this trial UC-MSCs were safe and well tolerated. Mesoblast Limited have reported, via press release, findings from a randomised, controlled trial of Remestemcel-L (allogeneic bone marrow-derived MSCs) in patients with COVID-19 ARDS. A reduction in day 60 mortality of 46% in patients under 65 with COVID 19 ARDS who received two intravenous doses of 2 million cells/kg 3 to 5 days apart is reported (26% vs 42%, hazard ratio 0.54, 95% CI (0.286, 1.005), *p* = 0.048) [36].

As a cellular product, MSC therapeutics derived from different sources and from different manufacturing techniques are considered unique and each requires robust evaluation in clinical trials. REALIST ORBCEL-C, the investigational medicinal product (IMP) in the REALIST trial, consists of human umbilical cord (UC)-derived CD362 enriched MSCs. Umbilical cord tissue (Wharton’s Jelly) provides a cost-efficient source of MSCs, utilising a tissue source that would otherwise be disposed of as a waste product and avoiding the potential risk of bone marrow harvesting. Anti-CD362 antibody enrichment of MSCs describes a technique of isolating MSCs, which enhances the homogeneity of the cellular product and is likely to fulfil future regulations regarding the production of ATMPs. In preclinical models, CD362-enriched MSCs are as effective as traditionally manufactured MSCs in attenuating lung injury [20].

The REALIST trial will conduct a phase 1 and phase 2 clinical trial of human umbilical cord-derived CD362-enriched MSCs (REALIST ORBCEL-C) in patients with moderate to severe ARDS, as defined by the Berlin criteria [1]. The hypothesis is that in patients with moderate to severe ARDS, human UC-derived CD362-enriched MSCs (REALIST ORBCEL-C) are safe and will improve important outcomes. In light of the COVID-19 pandemic, the REALIST trial will recruit an additional cohort of patients with COVID-19 ARDS during phase 2. Due to the clinical differences in patients with ARDS due to COVID-19 and other causes of ARDS, these will be recruited as separate cohorts [37]. A structured protocol summary has previously been published in *Trials* [38].

## Objectives {7}

The primary objective of the study is to assess the safety of a single intravenous infusion of REALIST ORBCEL-C cells in patients with moderate to severe ARDS.

The secondary objectives of the study are to determine, in patients with moderate to severe ARDS, the effect of a single intravenous infusion of REALIST ORBCEL-C cells on:

1. Physiological indices of respiratory dysfunction reflecting severity of ARDS, as measured by oxygenation index (OI), respiratory system compliance, and P/F ratio.
2. Sequential Organ Failure Assessment (SOFA) score.
3. Extubation and reintubation.
4. Ventilator-free days (VFDs) at day 28.
5. Duration of mechanical ventilation.
6. Length of ICU and hospital stay.
7. Twenty-eight-day and 90-day mortality.

## Trial design {8}

Phase 1 is a multicentre open-label dose-escalation pilot trial in which cohorts of patients with moderate to severe ARDS will receive increasing doses of a single infusion of REALIST ORBCEL-C in a 3 + 3 design [39]. Three cohorts with three subjects/cohort are planned. Planned doses for the three cohorts, in the absence of safety concerns, are 100 × 10^6^ cells, 200 × 10^6^ cells and 400 × 10^6^ cells. Patients will be assessed for dose-limiting toxicity, defined by any serious adverse event related to study drug administration, at day 7. If none of the three patients in the cohort experiences dose-limiting toxicity, then we will proceed to the next cohort. However, if one of the patients in the cohort shows dose-limiting toxicity, a further three subjects will be treated at the same dose level. The dose escalation will continue until at least two patients among a cohort of three to six patients have dose-limiting toxicity or until 400 × 10^6^ cell dose in three patients is achieved. If dose-limiting toxicity occurs, then the lower dose (maximal tolerated dose, MTD) will be used in the phase 2 trial.

**Fig. 1:**
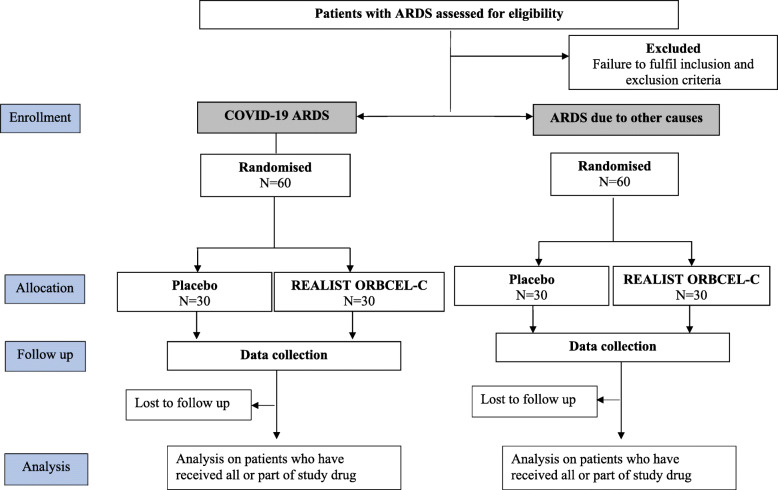
CONSORT diagram

Phase 2 is a multicentre randomised, triple-blind, allocation concealed placebo-controlled trial of REALIST ORBCEL-C in patients with moderate to severe ARDS. The phase 2 trial will recruit 2 separate cohorts of patients with ARDS due to COVID-19 and ARDS due to other causes. Within each cohort, patients will be randomised 1:1 to receive a single infusion of REALIST ORBCEL-C (400 × 10^6^ cells or maximum tolerated dose as determined by phase 1) or placebo (Fig. 1). To facilitate timely reporting, data from each cohort will be reported when the primary outcome is available.

## Methods: participants, interventions and outcomes

### Study setting {9}

Mechanically ventilated patients with moderate to severe ARDS, as defined by the Berlin criteria [1], will be recruited from participating intensive care units (ICUs) across the UK. A list of participating clinical sites is provided in a supplemental document (Supplemental file 1: List of participating sites).

### Eligibility criteria {10}

Eligibility criteria are outlined in Table 1. Inclusion and exclusion criteria are designed to include those who reflect a general population of critically ill patients with ARDS who may benefit from the therapeutic intervention and exclude patients more likely to experience an adverse reaction. Eligibility will be confirmed by a medically qualified person on the research team. Co-enrolment in other trials may be considered on a case-by-case basis in keeping with UK guidelines for co-enrolment in critical care trials [40].

**Table 1:** Inclusion and exclusion criteria

Inclusion criteria 1. Moderate to severe ARDS as defined by the Berlin definition a. Onset within 1 week of identified insult. b. Within the same 24-h time period i. Hypoxic respiratory failure (PaO_2_/FiO_2_ ratio ≤ 27 kPa on PEEP ≥ 5 cm H_2_0) ii. Bilateral infiltrates on chest X-ray consistent with pulmonary oedema not explained by another pulmonary pathology. iii. Respiratory failure not fully explained by cardiac failure or fluid overload. 2. The patient is receiving invasive mechanical ventilation. 3. In a separate COVID-19 cohort, COVID-19 based on clinical diagnosis or PCR test.	
Exclusion criteria 1. More than 72 h from the onset of ARDS* 2. Age < 16 years 3. Patient is known to be pregnant 4. Major trauma in prior 5 days 5. Presence of any active malignancy (other than non-melanoma skin cancer) that required treatment within the last year. 6. WHO Class III or IV pulmonary hypertension 7. Venous thromboembolism currently receiving anti-coagulation or within the past 3 months 8. Currently receiving extracorporeal life support (ECLS) 9. Severe chronic liver disease with Child-Pugh score > 12 10. DNAR (Do Not Attempt Resuscitation) order in place 11. Treatment withdrawal imminent within 24 h 12. Consent declined 13. Prisoners 14. Non-English speaking patients or those who do not adequately understand verbal or written information unless an interpreter is available 15. Previously enrolled in the REALIST trial	

*In phase 1, more than 48 h from the onset of ARDS

### Who will take informed consent? {26a}

Prospective informed consent will be sought by an appropriately trained member of the research team in keeping with the ethical principles of the Declaration of Helsinki and Good Clinical Practice (GCP) and according to the required legal framework (The Medicines for Human Use (Clinical Trials) Regulations, 2004). Patients are likely to be incapacitated due to the nature of the condition; therefore, informed consent will be sought from a patient representative, either a Personal Legal Representative (PerLR) who is usually the patient’s next of kin, or a Professional Legal Representative (ProLR), a doctor who is not connected with the conduct of the trial. If the PerLR is not available at the clinical site due to COVID-19 restrictions, a member of the research team may contact the PerLR by telephone and seek verbal agreement to participate in the trial. Verbal agreement is recorded on a telephone agreement form and must be signed by a second member of staff who has witnessed the telephone advice. Once patients regain capacity, consent to continue will be sought. Where it is not possible to get consent to continue face to face, patients will be telephoned to ask for consent to continue. Patients may withdraw or be withdrawn (by PerLR or ProLR) from the trial at any time without prejudice.

### Additional consent provisions for collection and use of participant data and biological specimens {26b}

Consent for collection, use and storage of participant data and biological specimens will be sought. Consent will be sought for biological samples to be stored indefinitely for use in this trial and for future use should new scientific research or techniques become available. This includes sharing of samples with external non-NHS organisations to undertake planned and future analysis, including genetic analysis, transfer abroad and commercial research. All samples will be anonymised.

### Interventions

#### Explanation for the choice of comparators {6b}

The comparator, Plasma-Lyte 148, has been chosen as the placebo as it is the diluent used in the cellular product.

#### Intervention description {11a}

*The Investigational Medicinal Products (IMP) are:*

1. Allogeneic donor anti-CD362 antibody enriched human UC-derived mesenchymal stromal cells (REALIST ORBCEL-C) supplied as a sterile, single-use cryopreserved cell suspension containing a fixed cell dose of either 100 × 10^6^, 200 × 10^6^ or 400 × 10^6^ cells in 10, 20 or 40 ml volumes respectively. The cellular product is thawed and diluted with Plasma-Lyte 148 to a total volume of 200mls for intravenous administration.
2. Plasma-Lyte 148 solution for infusion with an equivalent volume of 200 ml (Placebo).

The doses chosen to assess the safety of REALIST ORBCEL-C are based on pre-clinical data and are comparable to doses used in previous human clinical trials of MSCs [21]. To simplify cell manufacture and delivery of the intervention, doses of 100 × 10^6^, 200 × 10^6^ or 400 × 10^6^ cells have been chosen which equates to a range of approximately 1.4, 2.8 and 5.7 × 10^6^ cells/kg for a predicted body weight (PBW) of 70 kg. The phase 1 trial will determine the maximum tolerated dose which will be used in the phase 2 trial.

##### REALIST ORBCEL-C manufacture

The cellular product (REALIST ORBCEL-C) is harvested from umbilical cord tissue (UCT) of unrelated and human leukocyte antigen (HLA) unmatched healthy donors and manufactured to Good Manufacturing Practice (GMP) standards under an MHRA MIA (IMP) authorisation by the Cellular and Molecular Therapies Division of the National Health Service Blood and Transplant (NHSBT). Donated UCT is procured by NHSBT as the starting material under licence by the Human Tissue Authority in accordance with the Human Tissues and Cells Directive 2004/23/EC. All UCT donations are made with informed consent and the donor is tested for a range of markers of infection in accordance with guidance from the Advisory Committee on the Safety of Blood, Tissues and Organs [41]. Cells isolated from UCT are first enriched for CD362 prior to culture in flasks to passage 2 or 3. Cells are subsequently expanded to passage 4 (P4) with Terumo Quantum® cell expansion system. Cells harvested at P4 are re-suspended in Plasma-Lyte 148 buffer containing 2.5% Human Albumin Solution and an equal volume of CryoStor® CS10 cryoprotectant (DMSO 10%, BioLife Solutions, final DMSO concentration 5%) to achieve a concentration of cells of 10 × 10^6^ cells per ml. Cryopreserved drug product is stored at fixed cell doses of either 100 × 10^6^, 200 × 10^6^ or 400 × 10^6^ cells in 10, 20 or 40 ml volumes respectively. Final drug product release testing includes appearance, morphology, sterility testing, endotoxin testing, post thaw cell count, cell viability testing (minimum threshold > 70% post thaw) and flow cytometry to confirm cell phenotype fulfils criteria of MSCs described by the international society for cellular therapy (ISCT) (minimum of 90% positive for MSC markers (CD90, CD73 and CD105) and cell purity exceeding 95% (based on maximum values of 5% expression of markers CD45, CD34 and HLA-DR)) [42]. Cells also undergo mycoplasma analysis and karyotyping. Following manufacture, quality control testing and certification by a Qualified Person (QP), REALIST ORBCEL-C stock is distributed to clinical sites’ cell therapy facility (CTF) for storage until required.

##### IMP administration

On receipt of the trial prescription, the IMP (REALIST ORBCEL-C or placebo) will be prepared by the CTF. The IMP will be transported to the ICU at the clinical site for administration. To maintain blinding, care will be taken to ensure the placebo is not released earlier than the typical time needed for preparation of the cell therapy product from the frozen cellular drug substance. All patients will be administered chlorphenamine 10 mg by peripheral intravenous bolus prior to administration of the IMP. A single dose of the IMP will be administered intravenously, via a dedicated infusion line, over approximately 30 to 90 min. Patients will be monitored throughout the infusion and for 5 h following completion of the infusion by a member of the research team. The IMP will be administered through a standard blood component administration set, with a 200 micron in-line filter. The IMP will have a 6-h expiry date from the beginning of its preparation, which for the cellular product will be the beginning of the thaw process. Validation work has confirmed that a cell viability > 70% is maintained for at least 6 h following the thaw procedure. Both REALIST-ORBCEL C and Plasma-Lyte 148 placebo will be labelled with equivalent 6-h expiry dates to avoid risk of unblinding. The IMP infusion must be completed prior to this expiry.

#### Criteria for discontinuing or modifying allocated interventions {11b}

The IMP infusion may be terminated if one of the following occurs:

- Suspected IMP related adverse event
- IMP expiry
- Death or discontinuation of active treatment
- Request from PerLR or ProLR to withdraw patient from the trial
- Decision by the treating physician on safety grounds

#### Strategies to improve adherence to interventions {11c}

Patients will be continuously monitored throughout the IMP infusion. A study drug administration guideline will provide detailed information on drug administration and guidelines will be provided for management during study drug infusion. Data will be recorded regarding the date/time of commencing and completion of the infusion. If the IMP infusion is discontinued, the reason will be recorded.

#### Provisions for post-trial care {30}

Post-trial care will be according to standard critical care guidelines and at the discretion of the treating physician.

#### Relevant concomitant care permitted or prohibited during the trial {11d}

Other aspects of care will be according to standard critical care guidelines and at the discretion of the treating physician. Lung protective ventilation will be the standard of care with a tidal volume of 6 ml/kg PBW [8].

### Outcomes {12}

#### Primary outcome

The primary safety outcome is the incidence of serious adverse events (SAEs). The primary efficacy outcome is oxygenation index (OI) at day 7. OI is a physiological index of the severity of ARDS and measures both impaired oxygenation and the amount of mechanical support delivered. OI is independently predictive of mortality in patients with ARDS [43]. Day 7 has been chosen as it is expected this time interval will minimise the competing effects of death and extubation, while allowing a sufficient time interval for biological effect to occur. OI is calculated as (mean airway pressure (cmH_2_O) x FiO_2_ × 100)/PaO_2_(kPa).

#### Secondary outcomes

Secondary clinical outcomes will also be assessed including OI at days 4 and 14; physiological indices of pulmonary function (respiratory system compliance, driving pressure and PF ratio) on days 4, 7 and 14; organ failure as measured by the SOFA score on days 4, 7 and 14; extubation and reintubation; VFDs to day 28; duration of mechanical ventilation; length of ICU and hospital stay; and 28- and 90-day mortality.

#### Translational outcomes

In order to determine the potential mechanisms of action of MSCs, translational outcomes will include the biological effect of MSCs on pulmonary and systemic inflammatory responses, pulmonary and systemic indices of epithelial and endothelial function and injury, indices of coagulation, anti-HLA antibodies and cardiac function.

### Participant timeline {13}

A schedule of assessments is provided in Table 2.

**Table 2:** Schedule of assessments during trial period

	Day 0	Day 1	Days 2–3	Day 4	Days 5–6	Day 7	Days 8–13	Day 14	Days 15–28	Day 90 (± 14 days)	1 year (± 30 days)	2 years (± 30 days)
Eligibility assessment	X											
Informed consent	X											
Enrolment/randomisation	X											
Baseline data	X											
Daily data		X	X	X	X	X	X	X				
Chlorphenamine administration		X										
IMP administration		X										
Adverse events		X	X	X	X	X	X	X	X	X		
ECHO data (phase 2 only)	X			X								
BAL sampling (where possible)	X			X								
Blood sampling	X			X		X		X			X	X
Anti-HLA Ab	X								X			
Urine sampling	X			X		X		X				
Mortality									X	X	X	X
Medical event											X	X

### Sample size {14}

The phase 1 trial will recruit up to 18 participants.

The sample size for the phase 2 REALIST trial is 60 patients with ARDS due to COVID-19 and 60 patients with ARDS due to other causes. Due to the clinical differences in patients with ARDS due to COVID-19 and other causes of ARDS, these will be recruited as separate cohorts.

Although the primary focus of the phase 2 trial is safety, there is, however, power to detect a difference in physiological outcomes. The primary efficacy outcome measure will be the difference in oxygenation index (OI) between the ORBCEL-C- and placebo-treated groups at day 7. Based on our data from a recently completed clinical trial in ARDS, the mean (standard deviation; SD) OI at day 7 in patients with ARDS is 62 (51)  cmH_2_O/kPa [44]. To allow 1:1 recruitment (ORBCEL-C vs placebo), a sample size of 56 subjects will have 80% power at a two-tailed significance level of 0.05 using a two-sample *t*-test to detect a clinically significant difference of 39 cmH_2_O/kPa in OI between groups. In a previous phase 2 study of similar size, we have found that an intervention can demonstrate a change in OI of a similar magnitude confirming a treatment effect of this size can be achieved [44]. Although we anticipate few withdrawals or loss to follow-up, we have allowed for this in the sample size calculation. In previous UK multicentre studies in the critically ill, < 3% withdrew consent or were lost to follow-up and on this basis a conservative drop-out rate of 5% has been estimated [45]. Therefore, a total of 60 evaluable patients who have received study drug (30 patients in the ORBCEL-C and 30 in the placebo group) will be recruited into each cohort.

### Recruitment {15}

Mechanically ventilated patients will be prospectively screened daily at all participating sites to identify eligible patients. Reasons for non-recruitment will be recorded in the screening log, along with an anonymised minimal dataset (ICNARC Case Mix Programme number, age, sex, APACHE II score, worst PF ratio at time of assessment, reasons for non-enrolment and vital status). Recruitment (including reasons for non-enrolment of screened patients) will be monitored by the Trial Management Group (TMG) and the Data Monitoring and Ethics Committee (DMEC).

## Assignment of interventions: allocation

### Sequence generation {16a}

The randomisation sequence will be generated by the Northern Ireland Clinical Trials Unit (NICTU) statistician using NQuery Advisor using variable block sizes.

### Concealment mechanism {16b}

The randomisation sequence will be saved in a restricted section of the Trial Management Folder which can only be accessed by the NICTU statistician and not those who enrol or assign interventions.

### Implementation {16c}

In phase 1, participants will be allocated to receive either 100, 200 or 400 × 10^6^ cells of REALIST ORBCEL-C according to the dose-escalation protocol. As a safety precaution, only one patient across all sites can receive treatment at one time and no further patients should receive treatment in the 24 h following completion of REALIST ORBCEL-C infusion. This process of enrolment and confirmation of the dose allocation will be co-ordinated by the NICTU.

In phase 2, participants will be randomised to either REALIST ORBCEL-C or placebo control in a 1:1 ratio, stratified by recruitment centre and vasopressor use. Randomisation will be via an automated centralised 24-h telephone or web-based randomisation system (CHaRT, Centre for Healthcare Randomised Trials, University of Aberdeen) by an appropriately trained and delegated member of the research team.

## Assignment of interventions: blinding

### Who will be blinded {17a}

The investigator, treating physician, other members of the site research team and participants will be blinded. Cell therapy facility, clinical trials pharmacists and designated NICTU staff will be unblinded to facilitate preparation of the IMP and distribution of the IMP supply to sites. The unblinded individuals will keep the treatment information confidential and will not discuss or release information on treatment allocation to the patient, the investigator or other members of the research team. The infusion bag containing either cell product or placebo will be masked at preparation and administered through a masked infusion set.

### Procedure for unblinding if needed {17b}

The investigator or treating physician may unblind a participant’s treatment assignment in the case of an emergency, when knowledge of the study treatment is essential for the appropriate clinical management or welfare of the patient. Should a treating clinician require emergency unblinding, they should contact the centralised telephone randomisation system (CHaRT, Centre for Healthcare Randomised Trials, University of Aberdeen) and follow the trial specific unblinding guidelines.

## Data collection and management

### Plans for assessment and collection of outcomes {18a}

Data will be collected according to the schedule of assessments outlined in Table 2 by appropriately trained members of the research team and recorded on an electronic case report form (CRF).

Baseline data (day 0) will be collected in the 24 h prior to IMP administration and will include patient demographics, height, weight, PBW, date and time of ICU admission, date and time of onset of mechanical ventilation, date and time of consent and enrolment (phase 1) or randomisation (phase 2), aetiology of ARDS, APACHE II score, first qualifying PF ratio, worst PF ratio, Murray Lung injury score, determinants of the SOFA score, temperature, ventilation parameters, arterial blood gas parameters, oxygenation index (OI), use of adjunctive therapies and clinical laboratory assessments.

Physiological and ventilatory parameters, along with temperature and vasopressor doses, will be recorded immediately prior to IMP administration, every 15 min during infusion of the IMP and every hour for the 5 h following IMP administration. Daily data will be collected until day 14 (or death or ICU discharge if sooner). It will include determinants of the SOFA score, temperature, ventilation and arterial blood gas parameters, use of adjunctive therapies and renal replacement therapy, clinical laboratory assessments and fluid balance. Additionally, arterial blood gas parameters will be recorded every 12 h on days 2 and 3. During phase 2, echocardiography parameters including but not limited to ventricular size and function and tricuspid annular plane systolic excursion (TAPSE) will be collected where possible on day 4.

Additional clinical data collected will include extubation, re-intubation, duration of ventilation and duration of critical care and hospital stay. VFDs to day 28 are defined as the number of days from the time of initiating unassisted breathing to day 28 after study drug administration, assuming survival for at least 48 h after initiating unassisted breathing and continued unassisted breathing to day 28. Adverse events will be collected until day 90. After discharge from hospital, participant survival and significant medical events at 1 and 2 years post IMP administration will be determined by review of medical records or telephone contact with the patient or their GP.

Blood and urine samples will be collected and processed by trained members of the research team according to sample processing guidelines. Bronchoscopy and broncheoalveolar lavage (BAL) will be undertaken where possible, in accordance with previous descriptions [46, 47]. Samples will be transferred to Queen’s University Belfast for storage until analysis for translational outcomes is undertaken following completion the phase 1 and phase 2 trials. Blood for anti-HLA antibodies will be collected at baseline and day 28, where possible, and processed in an NHS clinical laboratory following completion of the trial.

### Plans to promote participant retention and complete follow-up {18b}

Patients will be informed of their participation in the trial by the responsible clinician or a member of the research team once they regain capacity to understand the details of the trial. After discharge, participants will be sent a note thanking them for their participation in the study and reminding them we will be back in contact for follow-up.

In the event of a request to withdraw from the study, the researcher will determine which elements of the trial are to be withdrawn from. In the event that the request is to withdraw from all elements of the study, only anonymised data recorded up to the point of withdrawal will be included in the study analysis. Consent will also be requested to use the samples collected to that point.

### Data management {19}

Data for individual patients will be collected by the site team and recorded in an electronic case report form (CRF). For routinely collected clinical data, the patient’s medical record will be the source document. Data will be processed by the NICTU Standard Operating Procedures (SOPs) and trial-specific Data Management Plan. Data queries will be generated electronically for site staff to clarify data or provide missing information. Trial monitoring procedures will check the accuracy of entries on the electronic CRF against the source documents, the adherence to the protocol, procedures and Good Clinical Practice. Quality control will be implemented by the NICTU in the form of SOPs, which are defined to encompass aspects of the clinical data management process and to ensure standardisation and adherence to International Conference of Harmonisation Good Clinical Practice (ICH-GCP) guidelines and regulatory requirements.

### Confidentiality {27}

Each patient enrolled in the study will be allocated a unique Participant Study Number, which will be used throughout the study for participant identification. In order to maintain confidentiality, all CRFs, questionnaires, study reports and communication regarding the study will identify the patients by their unique participant study number and initials only. Patient confidentiality will be maintained at every stage and will not be made publicly available to the extent permitted by the applicable laws and regulations.

### Plans for collection, laboratory evaluation and storage of biological specimens for genetic or molecular analysis in this trial/future use {33}

As detailed above consent will be sought for storage and use of biological samples, including for genetic analysis.

## Statistical methods

### Statistical methods for primary and secondary outcomes {20a}

For the phase 1 trial, no formal statistical analysis will be performed on safety data. The primary analysis will be descriptive and will focus on adverse events. The number of pre-specified cell infusion associated events will also be reported. Descriptive analysis of pulmonary and non-pulmonary organ function will also be undertaken. A final analysis and report of the phase 1 study is planned following the last patient’s 90 day follow-up. The 2 year follow-up data will be reported thereafter.

For the phase 2 trial, adverse events and prespecified cell infusion-associated events will be reported as for the phase 1 study in a descriptive analysis. For continuously distributed outcomes, differences between groups will be tested using independent samples *t*-tests and analysis of covariance with transformations of variables to normality if appropriate, or non- parametric equivalents. Chi-square tests (or Fisher’s exact tests) will be used for categorical variables. All statistical tests will be 2-sided and a *p*-value of 0.05 will be considered as statistically significant.

Correlations between changes in the biological markers measured and physiological and clinical outcomes will be assessed by appropriate graphical and statistical methods including Pearson’s (or Spearman’s) correlation coefficient.

A final analysis and report of each cohort in the phase 2 study is planned following the last patient’s 90-day follow-up in each cohort. The 2-year follow-up data will be reported thereafter.

### Interim analyses {21b}

No interim analysis is planned.

### Methods for additional analyses (e.g. subgroup analyses) {20b}

In phase 2, the primary analysis will be conducted on outcome data obtained from randomised participants who receive at least some of their randomly allocated treatment. Adjusted (age, PF Ratio, APACHE II and vasopressor use) analysis will be carried out to determine if there is a statistically significant difference between the ORBCEL-C and placebo groups. It is possible that some subjects may not receive the full treatment dose. Therefore, a secondary analysis will be undertaken on the population who receive the complete treatment dose. An additional analysis in the COVID-19 cohort will also be conducted on patients who have a confirmed PCR diagnosis of COVID-19. Even if an additional risk factor for ARDS co-exists, these patients will be included in the COVID-19 cohort. A patient will be analysed according to the cohort to which they were randomised. A priori defined subgroup analyses will be undertaken for the primary efficacy outcome and selected secondary outcomes (VFDs at day 28, and 28-day mortality) based on severity of inflammation (as measured by plasma CRP and ferritin) and PF ratio.

### Methods in analysis to handle protocol non-adherence and any statistical methods to handle missing data {20c}

Every effort will be made to minimise missing baseline and outcome data in this trial. The level and pattern of the missing data in the baseline variables and outcomes will be established by forming appropriate tables and the likely causes of any missing data will be investigated. This information will be used to determine whether the level and type of missing data has the potential to introduce bias into the analysis results for the proposed statistical methods, or substantially reduce the precision of estimates related to treatment effects. If necessary, these issues will be dealt with using multiple imputation or Bayesian methods for missing data as appropriate.

### Plans to give access to the full protocol, participant level-data and statistical code {31c}

The full protocol is available as a supplemental document (Supplemental file 2: Protocol v6.0 30.09.2020). The phase 1 and phase 2 Statistical Analysis Plan is available for public access at http://www.nictu.hscni.net/realist-trial-documents/ (last accessed 20 April 2020) and are available as supplemental document (Supplemental file 3: Phase 1 SAP, Supplemental file 4: Phase 2 SAP). Requests for data sharing will be reviewed on an individual basis by the Chief Investigator. The study will comply with the good practice principles for sharing individual participant data from publicly funded clinical trials [48] and data sharing will be undertaken in accordance with the required regulatory requirements and in the spirit of open research.

## Oversight and monitoring

### Composition of the coordinating centre and trial steering committee {5d}

The Trial Management Group (TMG) will be responsible for the day-to-day operational management of the trial. It will be chaired by the Chief Investigator and have representatives from the NICTU and other co-investigators/collaborators who provide trial specific expertise. The conduct of the trial will be overseen by a Trial Steering Committee (TSC) on behalf of the Sponsor/Funder. The majority of the TSC are independent members. It will include the Chief Investigator, 2 of the co-investigators and a group of experienced critical care clinicians and trialists, as well as a lay representative.

### Composition of the data monitoring committee, its role and reporting structure {21a}

An independent data monitoring and ethics committee (DMEC) will provide oversight of data monitoring, safety and ethical conduct during trial conduct. The DMEC will provide recommendations for dose escalation during phase 1 and review the maximum tolerated dose prior to proceeding to phase 2. During phase 2, the DMEC will be convened monthly initially, and frequency of meetings will be kept under review. No interim analysis is planned but the DMEC will be provided with interim information on recruitment, AE reporting and deaths from all causes at 28 days.

### Adverse event reporting and harms {22}

Patients recruited to the REALIST trial are already critically ill and as such many patients will experience events that are expected in this population (examples include transient hypoxaemia, agitation, delirium, organ failure, nosocomial infections, skin breakdown and gastrointestinal bleeding). These will not be reported as an adverse event unless considered to be related to the IMP or unexpectedly severe or frequent. The following prespecified adverse events occurring within 6 h of the start of the infusion will be collected: increase in vasopressor dose ( ≥ Norepinephrine 0.1 mcg/kg/min, ≥ epinephrine 0.1 mcg/kg/min, commencement of any other vasopressor); new ventricular tachycardia, ventricular fibrillation or asystole; new cardiac arrhythmia requiring cardioversion; hypoxaemia requiring an increase in FiO_2_ ≥ 0.2 and an increase in PEEP ≥5 to maintain SpO_2_ in target range; and clinical scenario consistent with transfusion incompatibility or transfusion-related infection. Additionally, the following pre-specified adverse events occurring within 24 h of the start of infusion will be collected: any death, any cardiac arrest and temperatures > 38.5 °C or an increase of ≥1 °C when temperature was > 38.5 °C prior to IMP administration.

Seriousness and causality of the adverse event will be determined by the investigator. As REALIST ORBCEL-C has not been administered to patients with ARDS, all adverse events related to IMP will be considered unexpected. Therefore, all serious adverse events (SAEs) related to the IMP (thereby a serious adverse reaction, SAR) will be a suspected unexpected serious adverse reaction (SUSAR). All SAEs, SAR/SUSARs should be reported to the NICTU within 24 h of the research team becoming aware of the event.

### Frequency and plans for auditing trial conduct {23}

Trial monitoring will be conducted regularly throughout the trial by the NICTU. The frequency and type of monitoring will be detailed in the monitoring plan and agreed by the sponsor. Audit activities will be completed during the trial as agreed by the sponsor.

### Plans for communicating important protocol amendments to relevant parties (e.g. trial participants, ethical committees) {25}

All protocol amendments will be undertaken in accordance with the regulatory requirements. Substantial changes to the protocol will require REC and MHRA approval prior to implementation, except when modification is needed to institute an urgent safety measure to maintain patient safety.

### Dissemination plans {31a}

Publications will be reported according to the CONSORT 2010 statement [49]. The phase 1 trial will be published on completion. Data from each cohort in the phase 2 trial will be published when data on the primary outcome is available. Long-term data and mechanistic data will also be reported although may form the basis of separate publications.

#### Patient and public involvement

Patients and public involvement (PPI) in the development of the trial proposal and protocol development has been sought. Mr Barry Williams, the PPI representative, is a member of the Trial Steering Committee and has provided guidance throughout the trial development as well as provided input in the development of patient facing materials prior to their approved use. On completion of the study, we will seek advice on dissemination of the results.

## Discussion

REALIST ORBCEL-C CD362 is an advanced therapy medicinal product (ATMP), consisting of UC derived CD362 enriched human MSCs. MSCs are a heterogenous cell population, traditionally isolated by plastic adherence, with minimal defining criteria [42]. In previous clinical trials, there has been considerable variation in manufacturing techniques utilised resulting in calls to better define manufacturing methods, provide clarification of phenotypical cell characteristics and develop potency assays [50–52]. REALIST aims to provide a defined UC-derived MSC product, with CD362 enrichment reducing heterogeneity of the cell product. REALIST ORBCEL-C was developed for administration by Orbsen Therapeutics Ltd., and subsequently, this technology was transferred from Orbsen Therapeutics Ltd to NHSBT facilities. REALIST ORBCEL-C is manufactured to Good Manufacturing Practice (GMP) standards and fulfils regulatory requirements for ATMPs. Each variation in manufacturing required validation studies and impacted on the production and quality control schedules. Data from the START trial indicated that washing the cryoprotectant off the final drug before administration led to greater loss of cell viability than previously recognised [22]. In this study, we therefore wished to avoid a wash step to remove the cryoprotectant but maintain cell viability in the lowest concentration of DMSO given reports DMSO may have potential adverse effects [53]. Furthermore, legislative changes introduced by the European pharmacopoeia mandated additional sterility testing.

The REALIST trial aimed to manufacture this novel therapeutic product within existing NHS infrastructure, namely the NHSBT. It is hoped that establishing manufacture within existing NHS infrastructure lends itself to large-scale production and distribution for future clinical trials and clinical use if effective. The NHSBT have considerable experience in the handling and manipulation of cell therapies for bone marrow and haematopoietic stem cell transplant patients. Their experience of cellular products has been advantageous throughout the development and manufacturing process of this cellular product and in the implementation of infrastructure required to facilitate the trial. The benefits of using existing infrastructure have been evident during the expansion of the phase 2 trial at the outset of the COVID-19 pandemic. Existing cell therapy facilities were utilised to increase the network of clinical sites involved in the trial, supporting expediated recruitment of the COVID-19 ARDS cohort. Furthermore, NHSBT were able to support increased cell manufacture for an additional cohort of patients through use of their facilities at a second manufacturing site.

In March 2020, phase 2 of the trial was ready to commence, with appropriate approvals and resources in place. However, COVID-19 was declared a pandemic on the 11th of March, and on the 19th of March 2020, the National Institute for Health Research (NIHR) issued guidance to pause any new or ongoing studies at NHS sites that were not nationally prioritised COVID-19 studies [54, 55]. As early reports of COVID-19 disease emerged, it had become evident ARDS was a leading cause of mortality [56, 57]. As a novel disease, MSCs had not been investigated in COVID-19; however, the rationale for MSCs as a potential therapy for ARDS caused by COVID-19 was similar to that for ARDS due to other causes. Indeed, MSCs had previously been investigated in models of ARDS due to other viral respiratory pathogens and demonstrated reparative and immunomodulatory properties [58, 59]. Consequently, an opportunity was recognised to rapidly repurpose the REALIST trial to recruit a cohort of patients with ARDS due to COVID-19. Given the potential for differences between the clinical, biomarker and outcome characteristics of COVID-19 ARDS to a heterogenous group of ARDS, a COVID-19 only cohort was recruited at this stage. As recruitment to this cohort progressed, simultaneously relevant approvals and resources were sought to recruit a second cohort of patients with ARDS due to other causes. There is sufficient evidence in the differences in COVID and non-COVID ARDS in terms of standard of care and outcomes to support recruitment to two separate cohorts [37]. From a methodological point of view, the decision to recruit two separate cohorts allows streamlining of trial processes and will facilitate timely reporting of the results from each cohort.

## Trial status

Phase 1 was commenced in January 2019 and completed recruitment in January 2020. The findings of the 90-day analysis and follow-up to 1 year have been published [60]. Follow-up to 2 years is ongoing. As discussed in March 2020, phase 2 was repurposed to recruit a cohort of patients with ARDS due to COVID-19. Additional funding for this COVID-19 cohort was sought and awarded (Health and Social Care Research and Development Division Needs-Led Research Award). Submissions were made to the Research Ethics Committee (REC) and MHRA to amend the REALIST protocol to a COVID-19 specific population (Protocol v4.0 23.03.2020). The protocol amendment was accepted by REC on the 27th of March 2020 and the MHRA on the 30th of March 2020. Urgent Public Health status was awarded by the NIHR on 2 April 2020 and the trial opened to recruitment, recruiting its first participant on the same day. Sites open to recruitment across the UK were expanded from 5 to 12 sites to expediate recruitment in the pandemic setting. During the first wave of the COVID-19 pandemic in the UK, 13 patients were recruited. Recruitment progressed during the second wave of COVID-19 in Autumn/Winter 2020, with the final patient recruited in December 2020, achieving a total recruitment of 60 patients. Analysis of day 90 data has been conducted and reporting is in progress. Longer term safety data will be analysed and reported separately. In order to proceed with a non-COVID ARDS cohort, funding and resources were required to cover additional cell therapy manufacturing costs (provided by Wellcome Trust) and a further protocol amendment was required. This protocol amendment has been approved by REC (13 October 2020), MHRA (11 October 2020) and Sponsor (18 March 2021) and the trial is now open for recruitment at sites across the UK. Protocol version in use is v6.0 30.09.2020. It is anticipated recruitment will continue until Spring 2023, with longer term follow-up continuing until Spring 2025.

## Supplementary Information

**Additional file 1: Supplemental file 1.** List of participating hospital sites

**Additional file 2: Supplemental file 2.** REALIST Protocol v6.0 30.09.2020

**Additional file 3: Supplemental file 3.** REALIST SAP Phase 1

**Additional file 4: Supplemental file 4.** REALIST SAP Phase 2

## Abbreviations

APACHE: Acute Physiology and Chronic Health Evaluation
ARDS: Acute respiratory distress syndrome
ATMP: Advanced therapy medicinal product
BAL: Bronchoalveolar lavage
BM: Bone marrow
CONSORT: Consolidated Standards of Reporting Trials
CI: Confidence intervals
CRF: Case report form
CTF: Cell therapy facility
DMEC: Data Monitoring and Ethics Committee
DMSO: Dimethyl sulfoxide
FiO_2_: Fractional content of inspired oxygen
GMP: Good Manufacturing Practice
HLA: Human leukocyte antigen
ICU: Intensive care unit
IMP: Investigational medicinal product
MAPC: Multipotent adult progenitor cell
MHRA: Medicines and Healthcare Products Regulatory Agency
MSC: Mesenchymal stromal cell
MTD: Maximum tolerated dose
NICTU: Northern Ireland Clinical Trials Unit
NHS: National Health Service
NHSBT: National Health Serve Blood and Transplant
O_2_: Oxygen
OI: Oxygenation index
PaCO_2_: Partial pressure carbon dioxide
PaO_2_: Partial pressure oxygen
PBW: Predicted body weight
PerLR: Personal Legal Representative
PEEP: Positive end-expiratory pressure
ProLR: Professional Legal Representative
REC: Research Ethics Committee
SOFA: Sequential Organ Failure Assessment
SPIRIT: Standard Protocol Items: Recommendations for Interventional Trials
UC: Umbilical cord
UCT: Umbilical cord tissue
VFD: Ventilator-free days
