# Supplementary material for: Repair of acute respiratory distress syndrome by stromal cell administration (REALIST): a structured study protocol for an open-label dose-escalation phase 1 trial followed by a randomised, triple-blind, allocation concealed, placebo-controlled phase 2 trial
Source: Trials. 2022 May 13;23:401. doi: 10.1186/s13063-022-06220-0 (PMC9099345; doi:10.1186/s13063-022-06220-0)
Supplement: Supplementary file 1 — Additional file 1: Supplemental file 1. List of participating hospital sites [file 13063_2022_6220_MOESM1_ESM.pdf]

Hospital sites participating in the study

Guy’s and St Thomas’ Hospital, Guys’ and Saint Thomas’s NHS Foundation Trust

Kings College Hospital, King’s College Hospital NHS Foundation Trust

University College Hospital, University College London Hospital NHS Foundation Trust

Heartlands, University Hospital Birmingham NHS Foundation Trust

Queen Elizabeth Hospital, University Hospital Birmingham NHS Foundation Trust

Belfast City Hospital, Royal Victoria Hospital, and Mater Infirmorum, Belfast Health and Social Care Trust

Liverpool Royal Infirmary, Liverpool and Broadgreen University Hospitals NHS Trust

Manchester Royal Infirmary, Manchester University NHS Foundation Trust

Southampton Hospital, University Hospital Southampton NHS Foundation Trust

Sunderland Royal Hospital, South Tyneside and Sunderland NHS Foundation Trust

Wythenshawe Hospital, Manchester University NHS Foundation Trust

Edinburgh Royal Infirmary

Aintree University Hospital, Liverpool University Hospitals NHS Foundation Trust.
