# Supplementary material for: Repair of acute respiratory distress syndrome by stromal cell administration (REALIST): a structured study protocol for an open-label dose-escalation phase 1 trial followed by a randomised, triple-blind, allocation concealed, placebo-controlled phase 2 trial
Source: Trials. 2022 May 13;23:401. doi: 10.1186/s13063-022-06220-0 (PMC9099345; doi:10.1186/s13063-022-06220-0)
Supplement: Supplementary file 4 — Additional file 4: Supplemental file 4. REALIST SAP Phase 2 [file 13063_2022_6220_MOESM4_ESM.pdf]

REALIST
Phase 2

Repair of Acute Respiratory Distress Syndrome by Stromal Cell Administration

Protocol Number:	16154DMcA-AS
EudraCT Number:	2017-000584-33
Ethics Reference Number:	18/NE/0006
ClinicalTrials.gov	NCT03042143

STATISTICAL ANALYSIS PLAN

Final 1.0 15/12/2020

Northern Ireland Clinical Trials Unit
1st Floor Elliott Dynes
The Royal Hospitals
Grosvenor Rd
Belfast
BT12 6BA
Tel:

Contacts

Professor Danny McAuley – Chief Investigator
Clíona McDowell – Head of Statistics
Christina Campbell – Trial Statistician

Email: info@nictu.hscni.net
This document and all preceding versions will be stored in the Trial Master File for this trial

Contents

STATISTICAL ANALYSIS PLAN 1
ABBREVIATIONS 4
1. Background and Design 6
2. Outcome measures 8
2.1 Primary outcome measure(s) 8
2.2 Secondary outcome measures 8
3. Data 9
3.1 CRF Forms and variables 9
3.2 Management of datasets 9
3.3 Data completion schedule 9
3.4 Data verification 10
3.5 Data coding 11
4. Definition of terms 11
5. Sample Size Calculations 12
6. RANDOMISATION AND BLINDING 12
6.1 Randomisation 12
6.2 Blinding and Allocation Concealment 13
7. Analysis Principles 13
8. Analysis Details 15
8.1 Recruitment and follow-up patterns 15
8.2 CONSORT Flow Diagram 15
8.3 Baseline Characteristics 16
8.4 Trial treatment 16
8.5 Trial Outcomes 16
8.6 Descriptive statistics of main clinical lab assessments over time (Baseline to Day 14) 17
8.7 Safety 17
9. Additional Information 18
9.1 Trial Steering Committee (TSC) 18
9.2 Data Monitoring and Ethics Committee (DMEC) 18
10. Signatures of Approval 20
Appendix 1: Example Draft Summary Tables 21

ABBREVIATIONS

Abbreviation / Acronym	Full Wording
ABG	Arterial Blood Gas
AE	Adverse Event
ALI	Acute Lung Injury
APACHE	Acute Physiology and Chronic Health Evaluation
AR	Adverse Reaction
ARDS	Acute Respiratory Distress Syndrome
ATMP	Advanced Therapeutic Medicinal Product
BAL	Bronchoalveolar Lavage
BHSCT	Belfast Health and Social Care Trust
BM	Bone Marrow
CI	Chief Investigator
CFU-F	Colony Forming Unit Fibroblasts
CMP	Case Mix Programme
CO_2	Carbon Dioxide
CONSORT	Controlled Standards of Reporting Trials
COVID-19	Novel coronavirus (2019)
CPAP	Continuous positive airway pressure
CRF	Case Report Form
Crs	Respiratory compliance
CRP	C-reactive protein
CTA	Clinical Trial Authorisation
CTIMP	Clinical Trial Investigational Medicinal Product
CTU	Clinical Trials Unit
CXR	Chest X-ray
DMEC	Data Monitoring and Ethics Committee
DLT	Dose Limiting Toxicity
DMP	Data Management Plan
DMSO	Dimethyl Sulfoxide
DNAR	Do Not Attempt Resuscitation
ECLS	Extracorporeal Life Support
ECMO	Extracorporeal Membrane Oxygenation
EKG	Electrocardiogram
ELISA	Enzyme-Linked Immunosorbent Assay
EudraCT	European Clinical Trials Database
FiO_2	Fraction of Inspired Oxygen
GP	General Practitioner
GCP	Good Clinical Practice
GMP	Good Manufacturing Practice
HLA Ab	Human Leukocyte Antigen Anti-bodies
HTA	Human Tissue Authority
IB	Investigator Brochure
ICH	International Conference on Harmonisation
ICNARC	Intensive Care National Audit & Research Centre
ICU	Intensive Care Unit

ISF	Investigator Site File
IV	Intravenous
LPS	Lipopolysaccharide
MDM	Monocyte-derived Macrophages
MHRA	Medicines and Healthcare products Regulatory Agency
MMP	Matrix metalloproteinases
MSC	Mesenchymal Stromal Cell
MTD	Maximal Tolerated Dose
NHS	National Health Service
NHSBT	National Health Service Blood and Transplant
NICTU	Northern Ireland Clinical Trials Unit
NETs	Neuroendocrine Tumors
NMBD	Neuromuscular Blocking Drugs
O2	Oxygen
OI	Oxygenation Index
$PaCO_2$	Partial Pressure of Carbon Dioxide in arterial blood
PaO_2	Partial Pressure of Oxygen in arterial blood
PBW	Predicted Body Weight
PerLR	Personal Legal Representative
PEEP	Positive End Expiratory Pressure
P/F ratio	PaO_2/FiO_2 ratio
PI	Principal Investigator
PIS	Patient Information Sheet
ProfLR	Professional Legal Representative
QUB	Queens University Belfast
RAGE	Receptor for Advanced Glycation Endproducts
REC	Research Ethics Committee
RR	Respiratory Rate
SAE	Serious Adverse Event
SAP	Statistical Analysis Plan
SAPS	Simplified Acute Physiology Score
SDV	Source Data Verification
SOFA	Sequential Organ Failure Assessment
SOPs	Standard Operating Procedures
SP-D	Surfactant Protein-D
SUSAR	Suspected Unexpected Serious Adverse Reaction
TMF	Trial Master File
TMG	Trial Management Group
TSC	Trial Steering Committee
UAR	Unexpected Adverse Reaction
VFD	Ventilator Free Days
WHO	World Health Organisation

1. BACKGROUND AND DESIGN

The hypothesis under investigation is that in young people (aged 16-17 years) and adult patients with moderate to severe ARDS, human umbilical cord derived CD362 enriched MSCs, (REALIST ORBCEL-C cells) are safe and improve important outcomes.

The aim of this study is to conduct a phase 2 clinical trial of human umbilical cord derived CD362 enriched MSCs, (REALIST ORBCEL-C cells), in patients with ARDS.

The primary objective is to assess the safety of a single intravenous infusion of REALIST ORBCEL-C cells in patients with ARDS .

The secondary objectives are in patients with moderate to severe ARDS to determine the effect of a single intravenous infusion of REALIST ORBCEL-C cells on:

1. Physiological indices of respiratory dysfunction reflecting severity of ARDS, as measured by oxygenation index (OI), respiratory compliance, and P/F ratio.
2. Sequential organ failure assessment (SOFA) score.
3. Extubation and reintubation.
4. Ventilation free days at day 28.
5. Duration of ventilation.
6. Length of ICU and hospital stay.
7. 28-day and 90-day mortality.

The phase 2 trial is a randomised, double-blind, allocation concealed placebo-controlled study of 400 $x10_6$ cell dose of REALIST ORBCEL-C in patients with moderate to severe ARDS (Figure 1).
The phase 2 trial will recruit patients with ARDS due to COVID-19 and other causes of ARDS. Due to the potential differences in patients with ARDS due to COVID-19, these patients will be recruited as a separate cohort.

In PICO terms:

1. **Population**
   Young people (aged 16-17 years) and adult patients with moderate to severe ARDS due to COVID-19 or other causes of ARDS
2. **Intervention**
   $400x10^6$ cell dose of REALIST ORBCEL-C
3. **Comparator**
   Placebo
4. **Outcome**
   Safety and physiological indices of efficacy

Figure 1: Flow diagram for the phase 2 trial

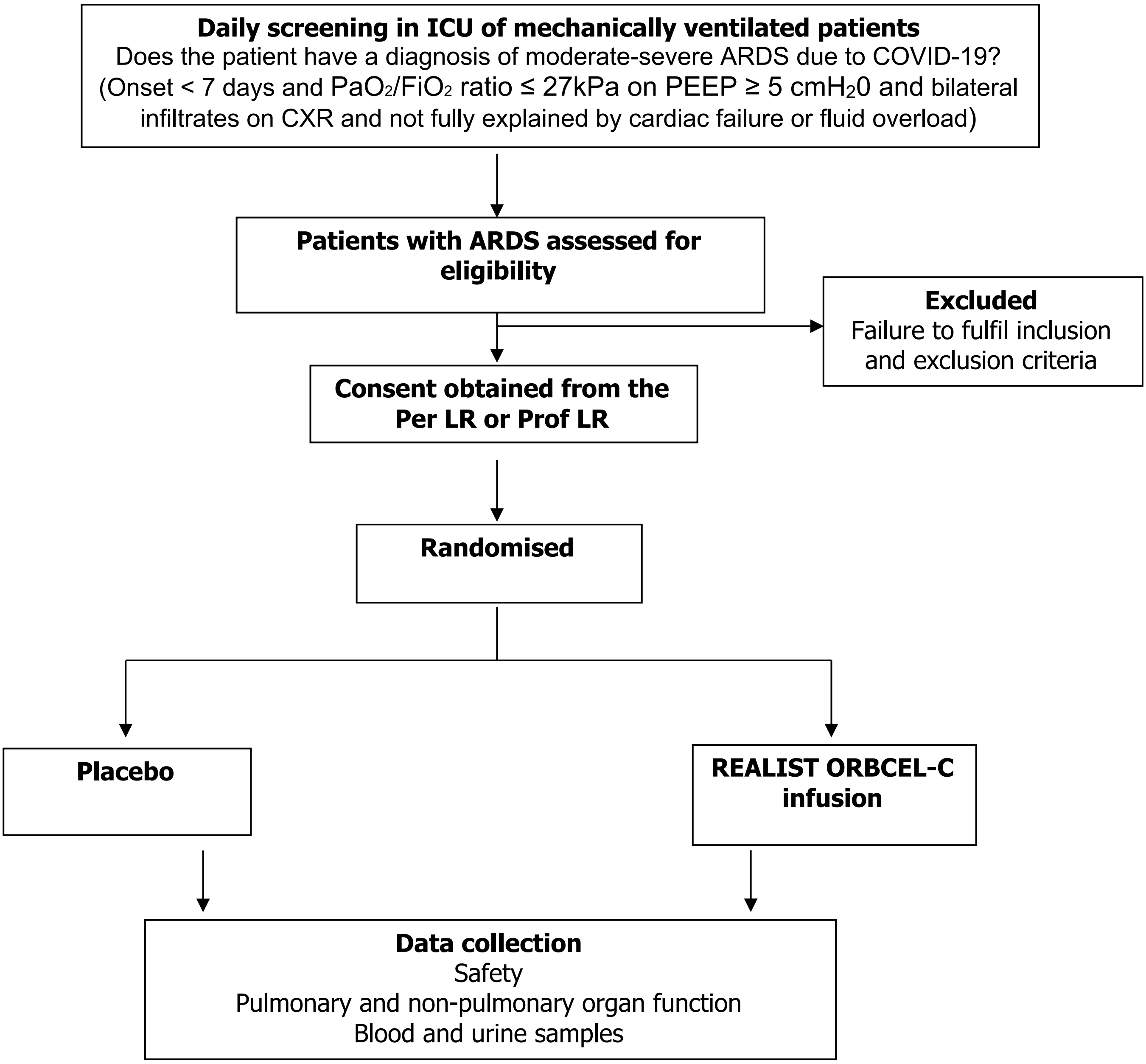

The overall duration of the study is 60 months. Patients will be followed up until 2 years post study drug administration.

Full details of the background to the trial and its design are presented in the protocol.

2. OUTCOME MEASURES

The phase 2 study will evaluate safety and efficacy outcomes in patients with ARDS due to COVID-19 or other causes of ARDS who have been administered MSC therapy. Although the primary focus of the phase 2 trial is safety, several outcomes will be evaluated to determine whether treatment with MSCs shows efficacy for important outcomes in patients with ARDS due to COVID-19 or other causes of ARDS.

2.1 Primary outcome measure(s)

The primary safety outcome is the incidence of serious adverse events (SAEs).

The primary efficacy outcome is oxygenation index (OI) at day 7.

OI is a physiological index of the severity of ARDS and measures both impaired oxygenation and the amount of mechanical ventilation delivered. OI is independently predictive of mortality in patients with ARDS. We have chosen day 7 as we expect this time interval will minimise the competing effects of death and extubation, while allowing a sufficient time interval for a biological effect to occur.

OI is calculated as (mean airway pressure (cm H_20) x FiO2 x 100) ÷ PaO_2 (kPa). These simple measurements are easily and routinely collected as part of standard ventilator practice.

2.2 Secondary outcome measures

The following secondary clinical outcomes will also be assessed:

1. OI at days 4 and 14.
2. Physiological indices of ARDS, as measured by respiratory compliance (Crs), driving pressure and P/F ratio on days 4, 7 and 14.
3. Organ failure as measured by the sequential organ failure assessment (SOFA) score on days 4, 7 and 14.
4. Extubation and reintubation.
5. Ventilation free days at day 28.
6. Duration of ventilation.
7. Length of ICU and hospital stay.
8. 28-day and 90-day mortality.

Outcomes will be measured at baseline and daily up to day 14 or until the patient is discharged from ICU or the patient dies, and thereafter as necessary for the 28-day and 90-day mortality. Patients will be followed up annually up to 2 years following recruitment.

3. DATA

3.1 CRF Forms and variables

Full details of the data to be collected and the timing of data collection are described in the trial protocol.

To ensure accurate, complete and reliable data are collected, the CTU will provide training to site staff in the format of investigator meetings and/or site initiation visits.

All data for an individual patient will be collected by the PI or designee and recorded in source documents/electronic CRF for the study. For routinely collected clinical data the NHS record will be the source document. Patient identification on the CRF will be through their unique participant study number, allocated at the time of enrolment. Data will be collected and recorded on the electronic CRF by the PI or designee as per the CRF submission guidelines.

If the participant is transferred to another hospital the PI or designated member of the site study team will liaise with the receiving hospital to ensure complete data capture as per CRF instruction. If this is not possible, the primary outcome must be collected as a minimum.

Data censorship for each trial participant will occur 90 days post study drug administration.

3.2 Management of datasets

Following the entry of patient data into the study database, the data will be processed as per the CTU Standard Operating Procedures (SOPs) and the study specific Data Management Plan (DMP). Data queries will be generated electronically for site staff to clarify data or request missing information. The designated site staff will be required to respond to these queries. All queries will be responded to/resolved within the study database and amended in the study database.

At the time of analysis:

- The Data Manager in collaboration with the Study Statistician will extract data from MACRO following procedures as detailed in the SOP DM09 Database Closure/Lock and the corresponding study Data Management Plan (DMP).

3.3 Data completion schedule

All patients recruited to the Phase 2 study must be evaluated according to the schedule of assessments as outlined in Table 3.3.1 Data will be collected at each of the following time points:

Table 3.3.1: Schedule of Assessments

	Day 0	Day 1	Day 2-3	Day 4	Day 5-6	Day 7	Day 8-13	Day 14	Day 15-28	Day 90 (+/- 14 days)	1 Year (+/- 30 days)	2 Year (+/- 30 days)
Eligibility assessment	X											
Informed consent	X											
Enrolment/ Randomisation	X											
Baseline data	X											
Daily data		X	X	X	X	X	X	X				
Chlorphenamine administration		X										
Study drug administration		X										
Adverse events		X	X	X	X	X	X	X	X	X		
ECHO data***	X			X								
Blood sampling[*,**]	X			X		X		X			X	X
Anti-HLA Ab[%]	X								X			
Urine sampling**	X			X		X		X				
Mortality[$]									X	X	X	X
Medical Event[#]											X	X

*Blood urine and bronchoalveolar lavage (BAL) samples for translational studies will be collected where possible
**Echocardiography data will be obtained where possible.
%Blood for anti-HLA Ab will be collected on day 0 and day 28 only where possible
$Mortality, including cause of death.
#Any significant medical event

3.4 **Data verification**

The CTU will provide training to site staff on trial processes and procedures including CRF completion and data collection.

On-site monitoring visits during the trial will check the accuracy of entries on the electronic CRF against the source documents, the adherence to the protocol, procedures and Good Clinical Practice (GCP).

Quality control is implemented by the CTU in the form of Standard Operating Procedures (SOPs), which are defined to encompass aspects of the clinical data management process, and to ensure standardisation and adherence to International Conference of Harmonisation Good Clinical Practice (ICH-GCP) guidelines and regulatory requirements.

Data validation will be implemented and discrepancy reports will be generated following data entry to identify discrepancies such as out of range, inconsistencies or protocol deviations based on data validation checks programmed in the clinical trial database.

A Data Monitoring & Ethics Committee (DMEC) will be convened for the study to carry out reviews of the study data at staged intervals during the study.

3.5 Data coding

The variable codings will be as specified on the CRF.

4. DEFINITION OF TERMS

Term	Definition
Ventilator Free Days (VFDs)	VFDs to day 28 are defined as the number of days from the time of initiating unassisted breathing to day 28 after study drug administration, assuming survival for at least 48 hours after initiating unassisted breathing and continued unassisted breathing to day 28. If a patient returns to assisted breathing and subsequently achieves unassisted breathing to day 28, VFDs will be counted from the end of the last period of assisted breathing to day 28. A period of assisted breathing lasting less than 24 hours and for the purpose of a surgical procedure will not count against the VFD calculation. If a patient was receiving assisted breathing at day 27 or dies prior to day 28, VFDs will be zero. Patients transferred to another hospital or other health care facility will be followed to day 28 to assess this endpoint.
Discharge	Discharge from critical care is defined as first discharge to a ward in the hospital or another hospital; a transfer between ICUs is not considered a discharge from critical care. Hospital discharge is the first date that the patient is discharged to home/community, a transfer between hospitals is not considered as a hospital discharge
Time to extubation	Time to extubation will be counted from time of study drug administration to extubation.
Duration of Ventilation	Duration of ventilation will be counted from time of study drug administration to being successfully free from assisted breathing.
Oxygenation Index	OI is calculated as (mean airway pressure (cm H_2O) x FiO2 x 100) ÷ PaO_2 (kPa).
Duration of stay	Duration of critical care and hospital stay will be counted from time of study drug administration to discharge.
Extubation	Extubation is defined as first time being successfully free from an endotracheal tube or a tracheostomy tube for 48hrs.
Unassisted breathing	Unassisted breathing i.e. no ventilatory support is defined as; extubated with supplemental oxygen or room air, or open T-tube

	breathing, or tracheostomy mask breathing, or CPAP without inspiratory pressure support for 48 hours. Patients receiving pressure support via non-invasive ventilation (except for sleep disordered breathing) or extra-corporeal lung support will be defined as receiving ventilatory support. The 48 hour period to define successful unassisted breathing is not included in the time to unassisted breathing.
Driving Pressure	Plateau Pressure – PEEP

5. SAMPLE SIZE CALCULATIONS

Although the primary focus of the phase 2 trial is safety, there is, however, power to detect a difference in physiological outcomes.

The sample size for the phase 2 REALIST trial is 60 patients with ARDS due to COVID-19 and 60 patients with ARDS not due to COVID-19 (30 in each of the ORBCEL-C and placebo groups). The recruitment, randomisation, treatment, outcome measurement and reporting, and follow-up processes will be as streamlined as possible.
Due to the clinical differences in patients with ARDS due to COVID-19 and other causes of ARDS, patients with ARDS due to COVID-19 and other causes of ARDS will be recruited as separate cohorts. This will also facilitate timely reporting of the results from each cohort.
The primary efficacy outcome measure will be the difference in oxygenation index (OI) between the ORBCEL-C and placebo treated groups at day 7. Based on our data from a recently completed clinical trial in ARDS, the mean (standard deviation; SD) OI at day 7 in patients with ARDS is 62(51)cmH2O/kPa . To allow 1:1 recruitment (ORBCEL-C vs placebo) a sample size of 56 subjects will have 80% power at a two-tailed significance level of 0.05 using a two-sample t-test to detect a clinically significant difference of 39 cmH2O/kPa in OI between groups. In a previous phase 2 study of similar size, we have found that an intervention can demonstrate a change in OI of a similar magnitude confirming a treatment effect of this size can be achieved. Although we anticipate few withdrawals or loss to follow-up we have allowed for this in the sample size calculation. In previous UK multicentre studies in the critically ill <3% withdrew consent or were lost to follow-up and on this basis a conservative drop-out rate of 5% has been estimated. Therefore a total of 60 evaluable patients who have received study drug (30 patients in the ORBCEL-C and 30 in the placebo group) will be recruited into each cohort.

6. RANDOMISATION AND BLINDING

6.1 Randomisation

Participants will be allocated to REALIST ORBCEL-C or placebo control in a 1:1 ratio. Randomisation will be stratified by recruitment centre and vasopressor use.

After informed consent, patients will be randomised via a centralised randomisation system. Sites will be provided with trial specific randomisation guidelines. Randomisation will be completed by an appropriately trained and delegated member of the research team. The randomisation sequence will be saved in a restricted section of the TMF, which can only be accessed by the trial statistician and not those who enrol or assign interventions. At the time of randomisation, each patient will be allocated a unique Participant Study Number, which will

be used throughout the study for participant identification. An entry will be recorded in the patients' medical notes noting enrolment into the study.

6.2 Blinding and Allocation Concealment

The cell therapy facility and clinical trials pharmacist will be unblinded. The unblinded individuals will keep the treatment information confidential and will not discuss or release information on treatment allocation to the patient, the investigator, or other members of the research team.

As in prior studies of MSCs, the infusion bag containing either the cell product or placebo will be masked at the time of preparation in the clinical site's cell therapy facility so that the contents of the infusion bag are not visible to the investigators or to the clinicians who are administering the study drug. The contents of the infusion bag will be administered through a masked infusion set.

The investigator or treating physician may unblind a participant's treatment assignment in the case of an emergency, when knowledge of the study treatment is essential for the appropriate clinical management or welfare of the patient. Should a treating clinician require emergency unblinding, they should contact the centralised allocation system and follow the trial specific unblinding guidelines. Unblinding will generate an email alert to the trial manager and CI. The date and reason for the unblinding must be recorded in the CRF.

7. ANALYSIS PRINCIPLES

Primary Efficacy Outcome (Oxygenation Index Day 7)

The primary analysis (unadjusted & using imputed values from last value carried forward) will be conducted on all outcome data obtained from all participants as randomised and who have received at least some of their randomly allocated treatment i.e. intention to treat. Adjusted (Age, PF Ratio, APACHE II and Vasopressor Use) analysis will be carried out to determine if there is a statistically significant difference between the Orbcel-C and placebo groups.

As it is possible that some subjects may not receive the full treatment dose, a secondary analysis will be undertaken on the population who receive the complete treatment dose i.e. per protocol.

A priori defined subgroup analyses will be undertaken for the primary efficacy outcome and selected secondary outcomes (VFDs at Day 28 and 28 Day Mortality) based on severity of inflammation as measured by plasma CRP, ferritin and PF Ratio. Subgroup analyses will be reported using 99% confidence intervals. Regression will be used with interaction terms (treatment group by subgroup) for the following subgroups:

1. Baseline CRP (<median, ≥median)
2. Baseline Ferritin(<1500, ≥1500)
3. Baseline PF Ratio (PaO2/FiO2) (< 20, ≥20)

Sensitivity analyses will assess the impact of missing data for the primary efficacy outcome by imputing extreme values (lowest and highest), mean substitution.

Primary Safety Outcome (incidence of serious adverse events (SAEs))

The number of SAEs and number (%) of patients experiencing the events will be reported. Fisher's exact test and proportion test will be used to check whether incidences of serious adverse events differ between the groups. Relative risk and 95% CI will be reported.

Secondary Outcomes

Adverse events and prespecified cell infusion associated events will be reported in a descriptive analysis.

For continuously distributed outcomes, differences between groups will be tested using independent samples t-tests and analysis of covariance with transformations of variables to normality if appropriate, or non-parametric equivalents

Chi-square tests (or Fisher's Exact tests if appropriate) will be used for categorical variables.

All statistical tests will be 2-sided and a p-value of 0.05 will be considered as statistically significant, unless adjustment for multiple testing was needed.

Correlations between changes in the biological markers measured and physiological and clinical outcomes will be assessed by appropriate graphical and statistical methods including Pearson's (or Spearman's) correlation coefficient.

Time-to-event outcomes will be analysed by survival methods and reported as hazard ratios with 95% CI.

An additional analysis in the COVID-19 cohort will also be conducted on patients who have a confirmed PCR diagnosis (at either baseline or discharge) of COVID-19 for all primary and secondary outcomes.

For the COVID-19 cohort even if an additional risk factor for ARDS co-exists, these patients will be included in the COVID-19 cohort. A patient will be analysed according to the cohort to which they were randomised.

A final analysis and report of each cohort in the phase 2 study is planned following the last patient's 90 day follow up in each cohort. All analyses will be performed separately for each cohort and for all patients combined.

The 2 year follow up data will be published thereafter and will be an important long term outcome.

All the power calculations and methodology for data analysis have been confirmed by the trial statistician from the Northern Ireland Clinical Trials Unit (NICTU).

Every effort will be made to minimise missing baseline and outcome data in this trial. The level and pattern of the missing data in the baseline variables and outcomes will be established by forming appropriate tables and the likely causes of any missing data will be investigated. This information will be used to determine whether the level and type of missing data has the potential to introduce bias into the analysis results for the proposed statistical methods, or substantially reduce the precision of estimates related to treatment effects.

8. ANALYSIS DETAILS

The results of the analyses will be reported following the principles of the ICH E3 guidelines on the Structure and Content of Clinical Study Reports.

8.1 Recruitment and follow-up patterns

- Recruitment by month, centre.
- Withdrawals by site

8.2 CONSORT Flow Diagram

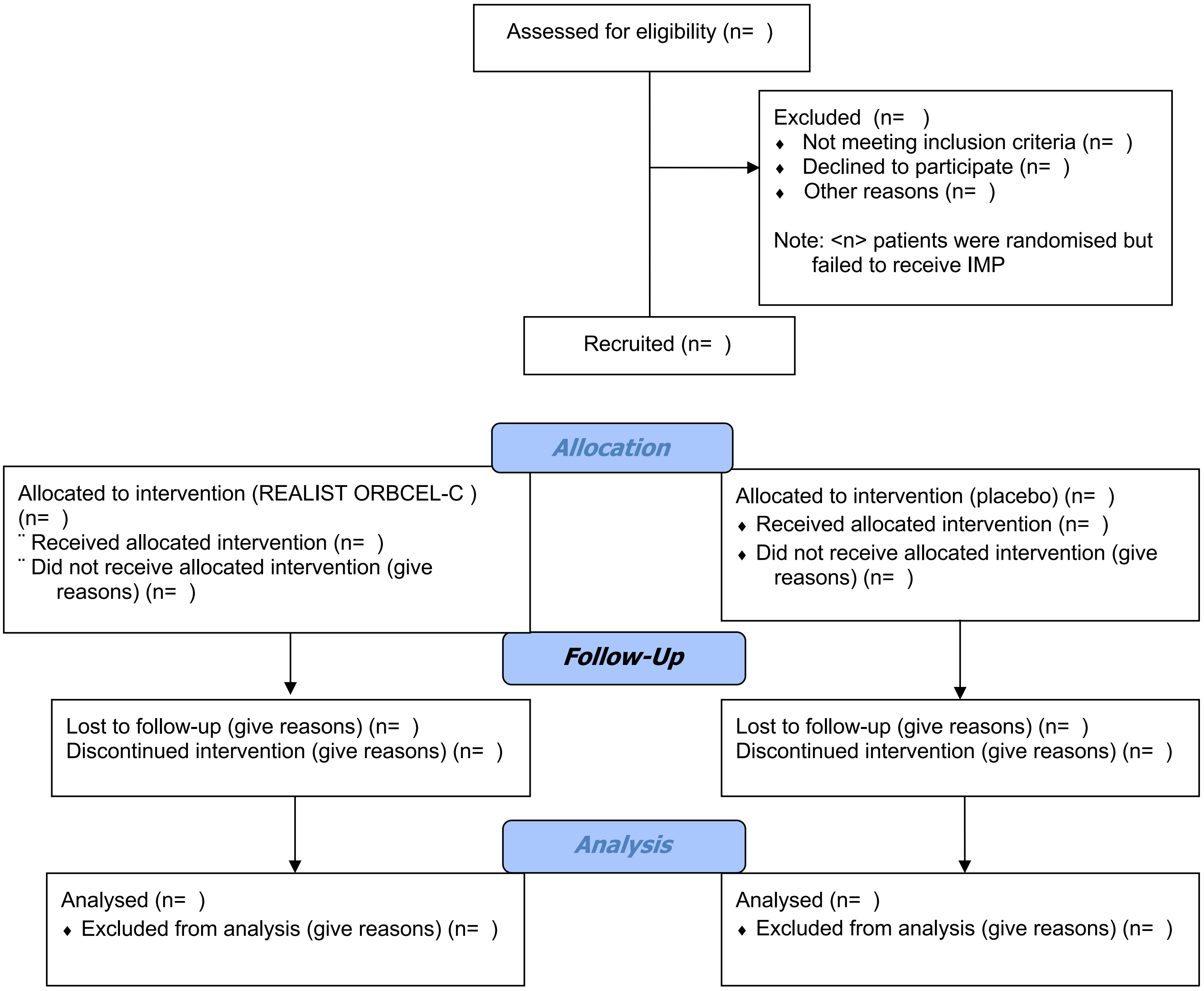

8.3 Baseline Characteristics

- Gender, n(%) by treatment arm
- Age (years), mean(sd) by treatment arm
- Weight (kg), mean(sd) by treatment arm
- Height (cm), mean(sd) by treatment arm
- Predicted body weight (kg), mean(sd) by treatment arm
- Temperature (°C) , mean(sd) by treatment arm
- Aetiology of ARDS, n(%) by treatment arm
- APACHE II Score, mean(sd) by treatment arm
- Murray Lung Injury Score (LIS) , mean(sd) by treatment arm
- First Qualifying P/F Ratio, mean(sd) by treatment arm
- Worst PaO2/FiO2 ratio (Day 0/24 hrs prior to randomisation), mean(sd) by treatment arm
- Total SOFA Score, mean(sd) by treatment arm
- Oxygenation Index, mean(sd) by treatment arm
- Lowest Mean Arterial Pressure (mmHg) , mean(sd) by treatment arm
- PEEP (cmH2O) , mean(sd) by treatment arm
- Plateau Pressure (cmH2O) , mean(sd) by treatment arm
- Driving Pressure (cmH2O), mean(sd) by treatment arm
- Mode of Ventilation, n(%) by treatment arm
- Tidal Volume (Vt) ml/kg PBW, mean(sd) by treatment arm
- COVID-19 diagnosis, n(%) by treatment arm
- CRP, mean(sd) by treatment arm
- Ferritin, mean(sd) by treatment arm
- Ethnicity, n(%) by treatment arm
- Vasopressor Use, n(%) by treatment arm
- Adjunctive Therapies as presented in Appendix 1, n(%) by treatment arm

Note: Gender, Age, Worst PaO2/FiO2 Ratio, APACHE II Score and vital status will also be presented for non-enrolled patients.

8.4 Trial treatment

- Study drug given, n(%) by treatment arm
- Full dose given, n(%) by treatment arm
- Reasons for termination of study drug, n(%) by treatment arm
- Protocol violations7, n(%) by treatment arm
- Post-randomisation withdrawal, n(%) by treatment arm
- Ineligible patient, n(%) by treatment arm
- Did not receive allocated treatment, n(%) by treatment arm
- Concomitant medications as presented in Appendix 1, n(%) by treatment arm
- Co-enrolment as presented in Appendix 1, n(%) by treatment arm

8.5 Trial Outcomes

- Primary safety outcome; incidence of SAEs, n(%) by treatment arm, risk ratio 95% CI
- Primary efficacy outcome; Oxygenation Index at day 7, mean(sd) by treatment arm, difference in mean & 95% CI
- Oxygenation Index (OI) at day 4 and day 14, mean(sd) by treatment arm, difference in mean & 95% CI

- Respiratory compliance at day 4, day 7 and day 14, mean(sd) by treatment arm, difference in mean & 95% CI
- P/F ratio at day 4, day 7 and day 14, mean(sd) by treatment arm, difference in mean & 95% CI
- Driving pressure at day 4, day 7 and day 14, mean(sd) by treatment arm, difference in mean & 95% CI
- Sequential Organ Failure Assessment (SOFA) score at day 4, day 7 and day 14, mean(sd) by treatment arm, difference in mean & 95% CI
- Extubations and reintubations
    - Time to 1st successful Extubation, mean(sd) by treatment arm and median(IQR) by treatment arm, hazard ratio 95% CI
    - Incidence of extubation, n(%) by treatment arm, risk ratio 95% CI
    - Incidence of reintubation, n(%) by treatment arm, risk ratio 95% CI
- Ventilation Free Days at day 28, mean(sd) by treatment arm, difference in mean & 95% CI
- Duration of Ventilation for both survivors and non-survivors, median(IQR) by treatment arm, p-value from Wilcoxon rank sum
- Length of ICU stay, median(IQR) by treatment arm, p-value from Wilcoxon rank sum
- Length of hospital stay, median (IQR) by treatment arm, p-value from Wilcoxon rank sum
- 28 day mortality , n(%) by treatment arm, risk ratio 95% CI
- 90 day mortality, n(%) by treatment arm, risk ratio 95% CI

8.6 Descriptive statistics of main clinical lab assessments over time (Baseline to Day 14)

- AST, mean(sd) by treatment arm
- ALT, mean(sd) by treatment arm
- ALP, mean(sd) by treatment arm
- CRP, mean(sd) by treatment arm
- PT, mean(sd) by treatment arm
- APTT, mean(sd) by treatment arm
- Fibrinogen, mean(sd) by treatment arm
- Hb, mean(sd) by treatment arm
- WBC, mean(sd) by treatment arm
- Neutrophils, mean(sd) by treatment arm
- Lowest eGFR, mean(sd) by treatment arm
- Highest Urea, mean(sd) by treatment arm
- Ferritin, mean(sd) by treatment arm

8.7 Safety

- Incidence of AEs, no. events (%) by treatment arm and system organ class, no. patients (%) by treatment arm and system organ class. Risk Ratio and 95% CI.
- Incidence of pre-specified AEs occurring within 6 hours of the start of infusion, no. events(%) by treatment arm and term, no. patients (%) by treatment arm and term. Risk Ratio and 95% CI.
- Incidence of pre-specified AEs occurring within 24 hours of the start of infusion, no. events(%) by treatment arm and term, no. patients (%) by treatment arm and term. Risk Ratio and 95% CI.
- Incidence of ARs, no. events (%) by treatment arm, no. patients (%) by treatment arm. Risk Ratio and 95% CI.

- Incidence of SAEs , no. events (%) by treatment arm and system organ class, no. patients (%) by treatment arm and system organ class. Risk Ratio and 95% CI.
- Incidence of SARs, no. events (%) by treatment arm, no. patients (%) by treatment arm. Risk Ratio and 95% CI.
- Incidence of SUSARs, no. events (%) by treatment arm, no. patients (%) by treatment arm. Risk Ratio and 95% CI.
- 7 day mortality, n(%) by treatment arm , risk ratio 95% CI
- 1 year mortality, n(%) by treatment arm , risk ratio 95% CI
- 2 year mortality, n(%) by treatment arm , risk ratio 95% CI

9. ADDITIONAL INFORMATION

9.1 Trial Steering Committee (TSC)

The conduct of the trial will be overseen by a Trial Steering Committee (TSC) on behalf of the Sponsor/Funder. The TSC will include the Chief Investigator (CI), 2 of the co-investigators and a group of experienced critical care clinicians and trialists as well as a "lay" representative. Annual meetings will be held, however as the Data Monitoring and Ethics Committee (DMEC) will meet during the phase 2 trial, the TSC may be convened to discuss issues and recommendations raised by the DMEC, in addition to the scheduled annual meetings. The roles and responsibilities of the TSC will be detailed in the Trial Steering Committee Charter. The TSC, in the development of this protocol and throughout the trial, will take responsibility for monitoring and guiding overall progress, scientific standards, operational delivery and protecting the rights and safety of trial participants. Meetings will be formally minuted and stored in the Trial Master File (TMF).

9.2 Data Monitoring and Ethics Committee (DMEC)

A Data Monitoring and Ethics Committee (DMEC) will be appointed comprising two clinicians with experience in undertaking clinical trials / caring for critically ill patients / cell therapy and a statistician who are independent of the trial. The DMEC will meet to agree conduct and remit, and the roles and responsibilities of the DMEC will be detailed in the Data Monitoring and Ethics Committee Charter. The DMEC will be convened monthly initially. Frequency of DMEC meetings should be kept under review by the DMEC and TSC. In the event of any safety concerns, additional unplanned DMEC meetings will be convened.

The DMEC's responsibility is to safeguard the interests of the trial participants, in particular with regard to safety, and assist and advise the TSC so as to protect the validity and credibility of the trial. The DMEC will monitor recruitment, adverse events and outcome data. During the recruitment period, reports will be provided to the DMEC which will include information on recruitment, AEs reported, and deaths from all causes at 28 and 90 days, along with any other data that the committee may request. The DMEC will advise the TSC on continuation of the trial. They will make recommendations to stop the trial for benefit on the basis of a sufficiently statistically significant benefit and an effect estimate that is sufficiently large to be likely to influence decisions about the use of the relevant therapy by clinicians outside of the trial. Meetings will be formally minuted and stored in the Trial Master File (TMF).

Following a recommendation from the DMEC, the TSC will decide what actions, if any, are required. It will be the responsibility of the TSC to inform the Sponsor if concerns exist about patient safety, following which the Sponsor will take appropriate action.

10. SIGNATURES OF APPROVAL

Date: 15/12/2020
Version: Final 1.0 15/12/2020
This document has completed a final review and is understood and approved by the following:

Danny McAuley	See attached email	See attached email
Chief Investigator Name	*Chief Investigator Signature*	Date dd/mm/yyyy
Clíona McDowell	See attached email	See attached email
Senior Statistician or designee Name	*Senior Statistician or designee Signature*	Date dd/mm/yyyy
Christina Campbell	See attached email	See attached email
Study Statistician Name	*Study Statistician Signature*	Date dd/mm/yyyy

APPENDIX 1: EXAMPLE DRAFT SUMMARY TABLES

Table x.x.x. Baseline Characteristics at trial entry

			Active N=	Placebo N=	Total
**Gender**	**Male**		n(%)	n(%)	n(%)
	**Female**		n(%)	n(%)	n(%)
**Age (years)**			xx.x (xx.x)	xx.x (xx.x)	xx.x (xx.x)
**Weight (kg)**			xx.x (xx.x)	xx.x (xx.x)	xx.x (xx.x)
**Height (cm)**			xx.x (xx.x)	xx.x (xx.x)	xx.x (xx.x)
**Predicted body weight (kg)**			xx.x (xx.x)	xx.x (xx.x)	xx.x (xx.x)
**Temperature (°C)**			xx.x (xx.x)	xx.x (xx.x)	xx.x (xx.x)
**Ethnicity**	**Caucasian**		n(%)	n(%)	n(%)
	**Black**		n(%)	n(%)	n(%)
	**Asian**		n(%)	n(%)	n(%)
	**Unknown**		n(%)	n(%)	n(%)
	**Other**		n(%)	n(%)	n(%)
**APACHE II Score**			xx.x (xx.x)	xx.x (xx.x)	xx.x (xx.x)
**Murray Lung Injury Score (LIS)**			xx.x (xx.x)	xx.x (xx.x)	xx.x (xx.x)
**First Qualifying P/F Ratio**			xx.x (xx.x)	xx.x (xx.x)	xx.x (xx.x)
**Worst PaO_2/FiO_2 ratio (Day 0/24 hrs prior to randomisation)**			xx.x (xx.x)	xx.x (xx.x)	xx.x (xx.x)
**Total SOFA Score**			xx.x (xx.x)	xx.x (xx.x)	xx.x (xx.x)
**Oxygenation Index**			xx.x (xx.x)	xx.x (xx.x)	xx.x (xx.x)
**Lowest Mean Arterial Pressure (mmHg)**			xx.x (xx.x)	xx.x (xx.x)	xx.x (xx.x)
**PEEP (cmH$_2$O)**			xx.x (xx.x)	xx.x (xx.x)	xx.x (xx.x)
**Plateau Pressure (cmH$_2$O)**			xx.x (xx.x)	xx.x (xx.x)	xx.x (xx.x)
**Driving Pressure (cmH$_2$O)**			xx.x (xx.x)	xx.x (xx.x)	xx.x (xx.x)
**Ferritin**			xx.x (xx.x)	xx.x (xx.x)	xx.x (xx.x)
**CRP**			xx.x (xx.x)	xx.x (xx.x)	xx.x (xx.x)
**Mode of Ventilation**	**SIMV**		n(%)	n(%)	n(%)
	**PS**		n(%)	n(%)	n(%)
	**Other**		n(%)	n(%)	n(%)
	**None**		n(%)	n(%)	n(%)
**Vasopressor Use**	**Yes**		n(%)	n(%)	n(%)
	**No**		n(%)	n(%)	n(%)
**Tidal Volume (Vt) ml/kg PBW**			xx.x (xx.x)	xx.x (xx.x)	xx.x (xx.x)
**COVID-19 Diagnosis**	**Clinical**		n(%)	n(%)	n(%)
	**Assay**		n(%)	n(%)	n(%)
**Adjunctive Therapies**	**Airway Pressure Release Ventilation (APRV)?**	**Yes**	n(%)	n(%)	n(%)
		**No**	n(%)	n(%)	n(%)
	**High-Frequency Oscillatory Ventilation (HFOV)**	**Yes**	n(%)	n(%)	n(%)
		**No**	n(%)	n(%)	n(%)
	**Neuromuscular Blocking Drugs (NMBD)**	**Yes**	n(%)	n(%)	n(%)
		**No**	n(%)	n(%)	n(%)
	**Nitric Oxide**	**Yes**	n(%)	n(%)	n(%)
		**No**	n(%)	n(%)	n(%)
	**Prone Position**	**Yes**	n(%)	n(%)	n(%)
		**No**	n(%)	n(%)	n(%)
	**Other**	**Yes**	n(%)	n(%)	n(%)
		**No**	n(%)	n(%)	n(%)

Mean (SD) (or median(IQR) if appropriate) presented for continuous variables and no. (%) for all categorical variables.

Table x.x.x. Treatment after Trial Entry

					Active	Placebo
					n = <n>	n = <n>
**Study drug given***					n(%)	n(%)
**Full dose given**					n(%)	n(%)
**Reasons for termination of study drug**	**Study drug related adverse event**				n(%)	n(%)
	**Study drug expiry**				n(%)	n(%)
	**Death or discontinuation of active treatment**				n(%)	n(%)
	**Request from PerLR or ProLR to withdraw the patient from the study**				n(%)	n(%)
	**Decision by the attending clinician on safety grounds**				n(%)	n(%)
**Protocol Violations**	**Eligibility**				n(%)	n(%)
	**Study Drug Administration**				n(%)	n(%)
	**SAE reporting**				n(%)	n(%)
	**Other**				n(%)	n(%)
**Post-randomisation withdrawal**					n(%)	n(%)
**Withdrawal from**	**Study drug administration**				n(%)	n(%)
	**Confirmation of vital status**				n(%)	n(%)
	**On-going data collection following hospital discharge**				n(%)	n(%)
	**Other elements of the REALIST trial**				n(%)	n(%)
**Did not receive allocated treatment** [a]					n(%)	n(%)
**Received treatment from other group** [b]					n(%)	n(%)
**Concomitant Medications (at any point during hospital stay)**	**Anti-viral Medications**	**Yes**	**Keletra**	**Yes**	n(%)	n(%)
				**No**	n(%)	n(%)
			**Remedesivir**	**Yes**	n(%)	n(%)
				**No**	n(%)	n(%)
			**Other**	**Yes**	n(%)	n(%)
				**No**	n(%)	n(%)
		**No**			n(%)	n(%)
	**Experimental Treatments**	**Yes**	**IL-6 blockade**	**Yes**	n(%)	n(%)
				**No**	n(%)	n(%)
			**IL-1 blockade**	**Yes**	n(%)	n(%)
				**No**	n(%)	n(%)
			**IFN**	**Yes**	n(%)	n(%)
				**No**	n(%)	n(%)
			**Macrolides**	**Yes**	n(%)	n(%)
				**No**	n(%)	n(%)
			**Convalescent Plasma**	**Yes**	n(%)	n(%)
				**No**	n(%)	n(%)
			**Heparin (no used for prevention/treatment of thrombosis)**	**Yes**	n(%)	n(%)
				**No**	n(%)	n(%)
			**Other**	**Yes**	n(%)	n(%)
				**No**	n(%)	n(%)
		**No**			n(%)	n(%)
	**Steroids**	**Yes**	**Hydrocortisone**	**Yes**	n(%)	n(%)
				**No**	n(%)	n(%)
			**Dexamethasone**	**Yes**	n(%)	n(%)
				**No**	n(%)	n(%)
			**Methylprednisolone**	**Yes**	n(%)	n(%)
				**No**	n(%)	n(%)

			**Prednisolone**	**Yes**	n(%)	n(%)
				**No**	n(%)	n(%)
			**Other**	**Yes**	n(%)	n(%)
				**No**	n(%)	n(%)
		**No**			n(%)	n(%)
**Co-Enrolment**	**Yes**	**Trial Name**	**Trial Arm**		n(%)	n(%)
	**No**				n(%)	n(%)

*Patients who have received at least some study drug
Mean (SD) (or median(IQR) if appropriate) presented for continuous variables and no. (%) for all categorical variables.
[a]- Numbers based on study drug administration data, [b]- Numbers based on the randomisation listing held by trial Statistician.

Table x.x.x Primary Efficacy Outcome (ITT Analyses)

Primary efficacy outcome; OI (cmH2O/kPa) at day 7 (intention to treat)	Unadjusted, Mean (SD)		Mean Difference (95% CI)	p-value	Adjusted, Mean (SD) †		Mean Difference (95% CI)	p-value
	Orbcel-C	Placebo			Orbcel-C	Placebo		
Observed values	xx.x (x.x)	xx.x (x.x)	xx.x (xx.x to xx.x)	0.xxx	xx.x (x.x)	xx.x (x.x)	xx.x (xx.x to xx.x)	0.xxx
**Imputed Values**								
Last value carried forward*	xx.x (x.x)	xx.x (x.x)	xx.x (xx.x to xx.x)	0.xxx	xx.x (x.x)	xx.x (x.x)	xx.x (xx.x to xx.x)	0.xxx
Minimum value	xx.x (x.x)	xx.x (x.x)	xx.x (xx.x to xx.x)	0.xxx	xx.x (x.x)	xx.x (x.x)	xx.x (xx.x to xx.x)	0.xxx
Maximum value	xx.x (x.x)	xx.x (x.x)	xx.x (xx.x to xx.x)	0.xxx	xx.x (x.x)	xx.x (x.x)	xx.x (xx.x to xx.x)	0.xxx
Mean substitution	xx.x (x.x)	xx.x (x.x)	xx.x (xx.x to xx.x)	0.xxx	xx.x (x.x)	xx.x (x.x)	xx.x (xx.x to xx.x)	0.xxx

*Primary Analysis
†adjusted for baseline age, PF Ratio, APACHE II and Vasopressor Use

Table x.x.x Primary efficacy Outcome (Per protocol Analyses*)

Primary efficacy outcome; OI (cmH2O/kPa) at day 7 (per protocol)	Unadjusted, Mean (SD)		Mean Difference (95% CI)	p-value	Adjusted, Mean (SD) †		Mean Difference (95% CI)	p-value
	Orbcel-C	Placebo			Orbcel-C	Placebo		
Observed values (n=<n>)	xx.x (x.x)	xx.x (x.x)	xx.x (xx.x to xx.x)	0.xxx	xx.x (x.x)	xx.x (x.x)	xx.x (xx.x to xx.x)	0.xxx
Imputed values(n=<n>)*	xx.x (x.x)	xx.x (x.x)	xx.x (xx.x to xx.x)	0.xxx	xx.x (x.x)	xx.x (x.x)	xx.x (xx.x to xx.x)	0.xxx

*Per protocol analyses includes only those patients who received IMP.
†adjusted for baseline age, PF Ratio, APACHE II and Vasopressor Use

Table x.x.x Primary efficacy Outcome Subgroup Analyses

Primary efficacy outcome; OI(Imputed values) (cmH2O/kPa) at day 7		Treatment Group		Mean Difference (99% CI)	Interaction Term
		Orbcel-C	Placebo		
CRP(U/L)	< median	xx.x (x.x)	xx.x (x.x)	xx.x (xx.x to xx.x)	0.xxx
	≥ median	xx.x (x.x)	xx.x (x.x)	xx.x (xx.x to xx.x)	
Ferritin (ng/ml)	<1500	xx.x (x.x)	xx.x (x.x)	xx.x (xx.x to xx.x)	0.xxx
	≥1500	xx.x (x.x)	xx.x (x.x)	xx.x (xx.x to xx.x)	
PF ratio (PaO2/FiO2)	<20	xx.x (x.x)	xx.x (x.x)	xx.x (xx.x to xx.x)	0.xxx
	≥20	xx.x (x.x)	xx.x (x.x)	xx.x (xx.x to xx.x)	

Table x.x.x Primary Outcome additional analyses

OI (cmH2O/kPa) at day 7	Active n=	Placebo n=	Difference (95% Confidence Intervals)	P-Value
Confirmed PCR COVID-19 diagnosis	xx.x (x.x)	xx.x (x.x)	xx.x (xx.x to xx.x)	0.xxx

Table x.x.x Secondary Outcomes

		Treatment Group		Mean Difference (95% CI)	p-value
		Orbcel-C	Placebo		
**Oxygenation Index ((OI) (cmH2O/kPa)) (Actual Values) †**					
**Day 4**		xx.x (xx.x)	xx.x (xx.x)	xx.x (xx.x to xx.x)	0.xxx
**Day 14**		xx.x (xx.x)	xx.x (xx.x)	xx.x (xx.x to xx.x)	0.xxx
**Respiratory compliance (ml/cmH2O) (Actual Values) †**					
**Day 4**		xx.x (xx.x)	xx.x (xx.x)	xx.x (xx.x to xx.x)	0.xxx
**Day 7**		xx.x (xx.x)	xx.x (xx.x)	xx.x (xx.x to xx.x)	0.xxx
**Day 14**		xx.x (xx.x)	xx.x (xx.x)	xx.x (xx.x to xx.x)	0.xxx
**Driving Pressure (cmH_2O) (Actual Values) †**					
**Day 4**		xx.x (xx.x)	xx.x (xx.x)	xx.x (xx.x to xx.x)	0.xxx
**Day 7**		xx.x (xx.x)	xx.x (xx.x)	xx.x (xx.x to xx.x)	0.xxx
**Day 14**		xx.x (xx.x)	xx.x (xx.x)	xx.x (xx.x to xx.x)	0.xxx
**PF ratio (PaO2/FiO2) (Actual Values) †**					
**Day 4**		xx.x (xx.x)	xx.x (xx.x)	xx.x (xx.x to xx.x)	0.xxx
**Day 7**		xx.x (xx.x)	xx.x (xx.x)	xx.x (xx.x to xx.x)	0.xxx
**Day 14**		xx.x (xx.x)	xx.x (xx.x)	xx.x (xx.x to xx.x)	0.xxx
**Sequential Organ Failure Assessment (SOFA) score (Actual Values) †**					
**Day 4**		xx.x (xx.x)	xx.x (xx.x)	xx.x (xx.x to xx.x)	0.xxx
**Day 7**		xx.x (xx.x)	xx.x (xx.x)	xx.x (xx.x to xx.x)	0.xxx
**Day 14**		xx.x (xx.x)	xx.x (xx.x)	xx.x (xx.x to xx.x)	0.xxx
**Time to 1st successful extubation (hours)***		xx.x (xx.x)	xx.x (xx.x)	xx.x (xx.x to xx.x)	0.xxx
**Incidence of extubation $ ****		n(%)	n(%)	xx.x (xx.x to xx.x)	0.xxx
**Incidence of reintubation $ ****		n(%)	n(%)	xx.x (xx.x to xx.x)	0.xxx
**Ventilation Free Days at day 28‡**		xx.x (xx.x, xx.x)	xx.x (xx.x, xx.x)		0.xxx
**Duration of Ventilation (Days)‡**	**All**	xx.x (xx.x, xx.x)	xx.x (xx.x, xx.x)		0.xxx
	**Survivors**	xx.x (xx.x, xx.x)	xx.x (xx.x, xx.x)		0.xxx
	**Non-Survivors**	xx.x (xx.x, xx.x)	xx.x (xx.x, xx.x)		0.xxx
**Length of ICU stay (Days)‡**		xx.x (xx.x, xx.x)	xx.x (xx.x, xx.x)		0.xxx

**Length of hospital stay (Days) ‡**	xx.x (xx.x, xx.x)	xx.x (xx.x, xx.x)		0.xxx
**28 day mortality ****	n(%)	n(%)	xx.x (xx.x to xx.x)	0.xxx
**90 day mortality ****	n(%)	n(%)	xx.x (xx.x to xx.x)	0.xxx

Mean (SD), median(IQR) or n(%) presented
*Hazard Ratio presented
$Number of patients with at least one occurrence.
†Mean (SD) presented for treatment arms and mean difference (95% CI), p-value from 2-sample t-test.
‡Median[IQR] and p-value from Wilcoxon rank sum presented
**Risk Ratio presented, p-value from chi-square (or fishers exact) presented.

Table x.x.x Subgroup Analyses for selected secondary outcomes

			**Treatment Group**		**Difference (99% CI)**	**Interaction Term**
			**Orbcel-C**	**Placebo**		
**Ventilation Free Days at day 28†**	**CRP(U/L)**	**< median**	xx.x (xx.x, xx.x)	xx.x (xx.x, xx.x)		0.xxx
		**≥ median**	xx.x (xx.x, xx.x)	xx.x (xx.x, xx.x)		
	**Ferritin (ng/ml)**	**<1500**	xx.x (xx.x, xx.x)	xx.x (xx.x, xx.x)		0.xxx
		**≥1500**	xx.x (xx.x, xx.x)	xx.x (xx.x, xx.x)		
	**PF ratio (PaO2/FiO2)**	**<20**	xx.x (xx.x, xx.x)	xx.x (xx.x, xx.x)		0.xxx
		**≥20**	xx.x (xx.x, xx.x)	xx.x (xx.x, xx.x)		
**28 day mortality ****	**CRP(U/L)**	**< median**	n(%)	n(%)	xx.x (xx.x to xx.x)	0.xxx
		**≥ median**	n(%)	n(%)	xx.x (xx.x to xx.x)	
	**Ferritin (ng/ml)**	**<1500**	n(%)	n(%)	xx.x (xx.x to xx.x)	0.xxx
		**≥1500**	n(%)	n(%)	xx.x (xx.x to xx.x)	
	**PF ratio (PaO2/FiO2)**	**<20**	n(%)	n(%)	xx.x (xx.x to xx.x)	0.xxx
		**≥20**	n(%)	n(%)	xx.x (xx.x to xx.x)	

†Median(IQR) presented for treatment arms
**Risk Ratio presented

Table x.x.x Descriptive statistics on main clinical lab assessments over time

	Active<br>n=	Placebo<br>n=
**AST (U/L)**		
**Day 0**	xx.x (xx.x)	xx.x (xx.x)
**Day 4**	xx.x (xx.x)	xx.x (xx.x)
**Day 7**	xx.x (xx.x)	xx.x (xx.x)
**Day 14**	xx.x (xx.x)	xx.x (xx.x)
**ALT (U/L)**		
**Day 0**	xx.x (xx.x)	xx.x (xx.x)
**Day 4**	xx.x (xx.x)	xx.x (xx.x)
**Day 7**	xx.x (xx.x)	xx.x (xx.x)
**Day 14**	xx.x (xx.x)	xx.x (xx.x)
**ALP (U/L)**		
**Day 0**	xx.x (xx.x)	xx.x (xx.x)
**Day 4**	xx.x (xx.x)	xx.x (xx.x)
**Day 7**	xx.x (xx.x)	xx.x (xx.x)
**Day 14**	xx.x (xx.x)	xx.x (xx.x)
**CRP (U/L)**		
**Day 0**	xx.x (xx.x)	xx.x (xx.x)
**Day 4**	xx.x (xx.x)	xx.x (xx.x)
**Day 7**	xx.x (xx.x)	xx.x (xx.x)
**Day 14**	xx.x (xx.x)	xx.x (xx.x)
**PT (s)**		
**Day 0**	xx.x (xx.x)	xx.x (xx.x)
**Day 4**	xx.x (xx.x)	xx.x (xx.x)
**Day 7**	xx.x (xx.x)	xx.x (xx.x)
**Day 14**	xx.x (xx.x)	xx.x (xx.x)
**APTT (s)**		
**Day 0**	xx.x (xx.x)	xx.x (xx.x)
**Day 4**	xx.x (xx.x)	xx.x (xx.x)
**Day 7**	xx.x (xx.x)	xx.x (xx.x)
**Day 14**	xx.x (xx.x)	xx.x (xx.x)
**Fibrinogen(g/l)**		
**Day 0**	xx.x (xx.x)	xx.x (xx.x)
**Day 4**	xx.x (xx.x)	xx.x (xx.x)
**Day 7**	xx.x (xx.x)	xx.x (xx.x)

**Day 14**	xx.x (xx.x)	xx.x (xx.x)
**Hb(g/L)**		
**Day 0**	xx.x (xx.x)	xx.x (xx.x)
**Day 4**	xx.x (xx.x)	xx.x (xx.x)
**Day 7**	xx.x (xx.x)	xx.x (xx.x)
**Day 14**	xx.x (xx.x)	xx.x (xx.x)
**WBC(x109/L)**		
**Day 0**	xx.x (xx.x)	xx.x (xx.x)
**Day 4**	xx.x (xx.x)	xx.x (xx.x)
**Day 7**	xx.x (xx.x)	xx.x (xx.x)
**Day 14**	xx.x (xx.x)	xx.x (xx.x)
**Neutrophils (x109/L)**		
**Day 0**	xx.x (xx.x)	xx.x (xx.x)
**Day 4**	xx.x (xx.x)	xx.x (xx.x)
**Day 7**	xx.x (xx.x)	xx.x (xx.x)
**Day 14**	xx.x (xx.x)	xx.x (xx.x)
**Highest Urea (mmol/L)**		
**Day 0**	xx.x (xx.x)	xx.x (xx.x)
**Day 4**	xx.x (xx.x)	xx.x (xx.x)
**Day 7**	xx.x (xx.x)	xx.x (xx.x)
**Day 14**	xx.x (xx.x)	xx.x (xx.x)
**Lowest eGFR (mL/min)**		
**Day 0**	xx.x (xx.x)	xx.x (xx.x)
**Day 4**	xx.x (xx.x)	xx.x (xx.x)
**Day 7**	xx.x (xx.x)	xx.x (xx.x)
**Day 14**	xx.x (xx.x)	xx.x (xx.x)
**Ferritin (ng/ml)**		
**Day 0**	xx.x (xx.x)	xx.x (xx.x)
**Day 4**	xx.x (xx.x)	xx.x (xx.x)
**Day 7**	xx.x (xx.x)	xx.x (xx.x)
**Day 14**	xx.x (xx.x)	xx.x (xx.x)

Table x.x.x. Safety by Treatment Group

		No. Events		No. Patients		RR (95% CI)	p-value
		Active	Placebo	Active	Placebo		
AEs, SAEs and SUSARs	Total AEs	n	n	n(%)	n(%)	xx.x (xx.x to xx.x)	0.xxx
	Related to study drug (AR)	n	n	n(%)	n(%)	xx.x (xx.x to xx.x)	0.xxx
	Total SAEs*	n	n	n(%)	n(%)	xx.x (xx.x to xx.x)	0.xxx
	Related to study drug (SAR)	n	n	n(%)	n(%)	xx.x (xx.x to xx.x)	0.xxx
	Related to study drug and unexpected (SUSAR)	n	n	n(%)	n(%)	xx.x (xx.x to xx.x)	0.xxx
SAEs	Cardiac Arrhythmia	n(%)	n(%)	n(%)	n(%)	xx.x (xx.x to xx.x)	0.xxx
	Cardiac General	n(%)	n(%)	n(%)	n(%)	xx.x (xx.x to xx.x)	0.xxx
	Gastrointestinal	n(%)	n(%)	n(%)	n(%)	xx.x (xx.x to xx.x)	0.xxx
	Etc…..	n(%)	n(%)	n(%)	n(%)	xx.x (xx.x to xx.x)	0.xxx
AEs	Cardiac Arrhythmia	n(%)	n(%)	n(%)	n(%)	xx.x (xx.x to xx.x)	0.xxx
	Cardiac General	n(%)	n(%)	n(%)	n(%)	xx.x (xx.x to xx.x)	0.xxx
	Gastrointestinal	n(%)	n(%)	n(%)	n(%)	xx.x (xx.x to xx.x)	0.xxx
	Etc…..	n(%)	n(%)	n(%)	n(%)	xx.x (xx.x to xx.x)	0.xxx
Pre-specified adverse events occurring within 6 hours of the start of infusion	An increase in vasopressor dose greater than or equal to the following:<br>a. Norepinephrine: 0.1 mcg/kg/min<br>b. Epinephrine: 0.1 mcg/kg/min<br>c. Commencement of any vasopressor including norepinephrine, epinephrine, vasopressin, phenylephrine, and dopamine	n(%)	n(%)	n(%)	n(%)	xx.x (xx.x to xx.x)	0.xxx
	New ventricular tachycardia, ventricular fibrillation or asystole	n(%)	n(%)	n(%)	n(%)	xx.x (xx.x to xx.x)	0.xxx
	New cardiac arrhythmia requiring cardioversion	n(%)	n(%)	n(%)	n(%)	xx.x (xx.x to xx.x)	0.xxx

		No. Events		No. Patients		RR (95% CI)	p-value
		Active	Placebo	Active	Placebo		
	**Hypoxemia requiring an increase in FiO2 of 0.2 or more and an increase in PEEP of 5 or more to maintain SpO2 in the target range**	n(%)	n(%)	n(%)	n(%)	xx.x (xx.x to xx.x)	0.xxx
	**Clinical scenario consistent with transfusion incompatibility or transfusion-related infection (e.g. urticaria, new bronchospasm).**	n(%)	n(%)	n(%)	n(%)	xx.x (xx.x to xx.x)	0.xxx
**Pre-specified adverse events occurring within 24 hours of the start of infusion**	**Any death**	n(%)	n(%)	n(%)	n(%)	xx.x (xx.x to xx.x)	0.xxx
	**Any cardiac arrest.**	n(%)	n(%)	n(%)	n(%)	xx.x (xx.x to xx.x)	0.xxx
	**Temperatures recorded as >38.5ºC or temperatures that are recorded as >38.5ºC prior to study drug administration and have increased by ≥1ºC.**	n(%)	n(%)	n(%)	n(%)	xx.x (xx.x to xx.x)	0.xxx

For no. of events %s are calculated within total AEs and SAEs respectively within treatment arm.
*Primary Safety Outcome

Table x.x.x. Additional Safety by Treatment Group

	Orbcel-C	Placebo	**RR (95% CI)**	**p-value**
**7 day mortality ****	n(%)	n(%)	xx.x (xx.x to xx.x)	0.xxx
**1 year mortality ****	n(%)	n(%)	xx.x (xx.x to xx.x)	0.xxx
**2 year mortality****	n(%)	n(%)	xx.x (xx.x to xx.x)	0.xxx

******Risk Ratio presented, p-value from chi-square (or fishers exact) presented.

Table x.x.x Additional Primary Efficacy Outcome Analyses (patients with confirmed PCR diagnosis of COVID-19) (COVID-19 Cohort only)

Primary efficacy outcome; OI (cmH2O/kPa) at day 7 (intention to treat)	Unadjusted, Mean (SD)		Mean Difference (95% CI)	p-value	Adjusted, Mean (SD) †		Mean Difference (95% CI)	p-value
	Orbcel-C	Placebo			Orbcel-C	Placebo		
Observed values	xx.x (x.x)	xx.x (x.x)	xx.x (xx.x to xx.x)	0.xxx	xx.x (x.x)	xx.x (x.x)	xx.x (xx.x to xx.x)	0.xxx
**Imputed Values**								
Last value carried forward*	xx.x (x.x)	xx.x (x.x)	xx.x (xx.x to xx.x)	0.xxx	xx.x (x.x)	xx.x (x.x)	xx.x (xx.x to xx.x)	0.xxx
Minimum value	xx.x (x.x)	xx.x (x.x)	xx.x (xx.x to xx.x)	0.xxx	xx.x (x.x)	xx.x (x.x)	xx.x (xx.x to xx.x)	0.xxx
Maximum value	xx.x (x.x)	xx.x (x.x)	xx.x (xx.x to xx.x)	0.xxx	xx.x (x.x)	xx.x (x.x)	xx.x (xx.x to xx.x)	0.xxx
Mean substitution	xx.x (x.x)	xx.x (x.x)	xx.x (xx.x to xx.x)	0.xxx	xx.x (x.x)	xx.x (x.x)	xx.x (xx.x to xx.x)	0.xxx

†adjusted for age, PF Ratio, APACHE II and Vasopressor Use

Table x.x.x Additional Primary Safety Outcome Analyses (patients with confirmed PCR diagnosis of COVID-19) (COVID-19 Cohort only)

	No. Events		No. Patients		RR (95% CI)	p-value
	Active	Placebo	Active	Placebo		
Total SAEs	n	n	n(%)	n(%)	xx.x (xx.x to xx.x)	0.xxx

Table x.x.x Additional Secondary Outcome Analyses (patients with confirmed PCR diagnosis of COVID-19) (COVID-19 Cohort only)

	Treatment Group		Mean Difference (95% CI)	p-value
	Orbcel-C	Placebo		
**Oxygenation Index ((OI) (cmH2O/kPa)) (Actual Values)**				
Day 4	xx.x (xx.x)	xx.x (xx.x)	xx.x (xx.x to xx.x)	0.xxx
Day 14	xx.x (xx.x)	xx.x (xx.x)	xx.x (xx.x to xx.x)	0.xxx
**Respiratory compliance (ml/cmH2O) (Actual Values)**				
Day 4	xx.x (xx.x)	xx.x (xx.x)	xx.x (xx.x to xx.x)	0.xxx
Day 7	xx.x (xx.x)	xx.x (xx.x)	xx.x (xx.x to xx.x)	0.xxx
Day 14	xx.x (xx.x)	xx.x (xx.x)	xx.x (xx.x to xx.x)	0.xxx
**Driving Pressure (cmH_2O) (Actual Values)**				
Day 4	xx.x (xx.x)	xx.x (xx.x)	xx.x (xx.x to xx.x)	0.xxx
Day 7	xx.x (xx.x)	xx.x (xx.x)	xx.x (xx.x to xx.x)	0.xxx
Day 14	xx.x (xx.x)	xx.x (xx.x)	xx.x (xx.x to xx.x)	0.xxx
**PF ratio (PaO2/FiO2) (Actual Values)**				
Day 4	xx.x (xx.x)	xx.x (xx.x)	xx.x (xx.x to xx.x)	0.xxx

		Treatment Group		Mean Difference (95% CI)	p-value
		Orbcel-C	Placebo		
**Day 7**		xx.x (xx.x)	xx.x (xx.x)	xx.x (xx.x to xx.x)	0.xxx
**Day 14**		xx.x (xx.x)	xx.x (xx.x)	xx.x (xx.x to xx.x)	0.xxx
**Sequential Organ Failure Assessment (SOFA) score (Actual Values)**					
**Day 4**		xx.x (xx.x)	xx.x (xx.x)	xx.x (xx.x to xx.x)	0.xxx
**Day 7**		xx.x (xx.x)	xx.x (xx.x)	xx.x (xx.x to xx.x)	0.xxx
**Day 14**		xx.x (xx.x)	xx.x (xx.x)	xx.x (xx.x to xx.x)	0.xxx
**Time to 1st successful extubation (hours)***		xx.x (xx.x)	xx.x (xx.x)	xx.x (xx.x to xx.x)	0.xxx
**Incidence of extubation $ ****		n(%)	n(%)	xx.x (xx.x to xx.x)	0.xxx
**Incidence of reintubation $ ****		n(%)	n(%)	xx.x (xx.x to xx.x)	0.xxx
**Ventilation Free Days at day 28‡**		xx.x (xx.x, xx.x)	xx.x (xx.x, xx.x)		0.xxx
**Duration of Ventilation (Days) ‡**	**All**	xx.x (xx.x, xx.x)	xx.x (xx.x, xx.x)		0.xxx
	**Survivors**	xx.x (xx.x, xx.x)	xx.x (xx.x, xx.x)		0.xxx
	**Non-Survivors**	xx.x (xx.x, xx.x)	xx.x (xx.x, xx.x)		0.xxx
**Length of ICU stay (Days) ‡**		xx.x (xx.x, xx.x)	xx.x (xx.x, xx.x)		0.xxx
**Length of hospital stay (Days)‡**		xx.x (xx.x, xx.x)	xx.x (xx.x, xx.x)		0.xxx
**28 day mortality ****		n(%)	n(%)	xx.x (xx.x to xx.x)	0.xxx
**90 day mortality ****		n(%)	n(%)	xx.x (xx.x to xx.x)	0.xxx

Mean (SD), median(IQR) or n(%) presented
*Hazard Ratio presented
$Number of patients with at least one occurrence.
†Mean (SD) presented for treatment arms and mean difference (95% CI), p-value from 2-sample t-test.
‡Median[IQR] and p-value from Wilcoxon rank sum presented
**Risk Ratio presented, p-value from chi-square (or fishers exact) presented.
